# Serial Sampling of Serum Protein Biomarkers for Monitoring Human Traumatic Brain Injury Dynamics: A Systematic Review

**DOI:** 10.3389/fneur.2017.00300

**Published:** 2017-07-03

**Authors:** Eric Peter Thelin, Frederick Adam Zeiler, Ari Ercole, Stefania Mondello, András Büki, Bo-Michael Bellander, Adel Helmy, David K. Menon, David W. Nelson

**Affiliations:** ^1^Division of Neurosurgery, Department of Clinical Neurosciences, University of Cambridge, Cambridge Biomedical Campus, Cambridge, United Kingdom; ^2^Department of Clinical Neuroscience, Karolinska Institutet, Stockholm, Sweden; ^3^Division of Anaesthesia, Department of Medicine, University of Cambridge, Cambridge, United Kingdom; ^4^Department of Surgery, Rady Faculty of Health Sciences, University of Manitoba, Winnipeg, MB, Canada; ^5^Clinician Investigator Program, Rady Faculty of Health Sciences, University of Manitoba, Winnipeg, MB, Canada; ^6^Department of Biomedical and Dental Sciences and Morphofunctional Imaging, University of Messina, Messina, Italy; ^7^Szentagothai Research Centre, University of Pecs, Pecs, Hungary; ^8^Department of Neurosurgery, University of Pecs, Pecs, Hungary; ^9^MTA-PTE Clinical Neuroscience MR Research Group, Pecs, Hungary; ^10^Wolfson Brain Imaging Centre, Department of Clinical Neurosciences, University of Cambridge, Cambridge Biomedical Campus, Cambridge, United Kingdom; ^11^Section of Perioperative Medicine and Intensive Care, Department of Physiology and Pharmacology, Karolinska Institutet, Stockholm, Sweden

**Keywords:** S100B, neuron-specific enolase, glial fibrillary acidic protein, ubiquitin carboxy-terminal hydrolase L1, neurofilament light, serum, biomarkers, traumatic brain injury

## Abstract

**Background:**

The proteins S100B, neuron-specific enolase (NSE), glial fibrillary acidic protein (GFAP), ubiquitin carboxy-terminal hydrolase L1 (UCH-L1), and neurofilament light (NF-L) have been serially sampled in serum of patients suffering from traumatic brain injury (TBI) in order to assess injury severity and tissue fate. We review the current literature of serum level dynamics of these proteins following TBI and used the term “effective half-life” (*t*_1/2_) in order to describe the “fall” rate in serum.

**Materials and methods:**

Through searches on EMBASE, Medline, and Scopus, we looked for articles where these proteins had been serially sampled in serum in human TBI. We excluded animal studies, studies with only one presented sample and studies without neuroradiological examinations.

**Results:**

Following screening (10,389 papers), *n* = 122 papers were included. The proteins S100B (*n* = 66) and NSE (*n* = 27) were the two most frequent biomarkers that were serially sampled. For S100B in severe TBI, a majority of studies indicate a *t*_1/2_ of about 24 h, even if very early sampling in these patients reveals rapid decreases (1–2 h) though possibly of non-cerebral origin. In contrast, the *t*_1/2_ for NSE is comparably longer, ranging from 48 to 72 h in severe TBI cases. The protein GFAP (*n* = 18) appears to have *t*_1/2_ of about 24–48 h in severe TBI. The protein UCH-L1 (*n* = 9) presents a *t*_1/2_ around 7 h in mild TBI and about 10 h in severe. Frequent sampling of these proteins revealed different trajectories with persisting high serum levels, or secondary peaks, in patients with unfavorable outcome or in patients developing secondary detrimental events. Finally, NF-L (*n* = 2) only increased in the few studies available, suggesting a serum availability of >10 days. To date, automated assays are available for S100B and NSE making them faster and more practical to use.

**Conclusion:**

Serial sampling of brain-specific proteins in serum reveals different temporal trajectories that should be acknowledged. Proteins with shorter serum availability, like S100B, may be superior to proteins such as NF-L in detection of secondary harmful events when monitoring patients with TBI.

## Introduction

Globally, traumatic brain injury (TBI) is one of the leading causes of death and disability among young adults, and due to sociodemographic changes, it is increasing among the elderly (1–3). TBI consists of two processes: the initial traumatic impact at the scene causing primary damage to the cerebral parenchyma and blood vessels, which can be followed by the onset of detrimental secondary insults (4), characterized by progressive cell death due to inflammation, impaired cerebral blood flow and metabolic function (5). As cells in the central nervous system are injured/compromised or succumb, they either secrete, release, or leak proteins, some of which are relatively enriched in the CNS (6). By measuring these proteins it is possible to assess the extent of cellular injury. Unconscious patients suffering from TBI are often treated in specialized neurointensive care units (NICU) where the goal is to detect, avoid, and treat these secondary insults to optimize cerebral recovery. Implementing measurement of these proteins of tissue fate (“biomarkers”) into clinical practice may help in the detection of secondary injury (7, 8).

The most studied TBI biomarker is S100B (9), a predominantly intracellular-, calcium-binding protein present primarily in mature, perivascular astrocytes (10). Other brain-specific proteins that have been extensively studied in TBI include the glycolytic enzyme neuron-specific enolase (NSE) (11); the astrocytic cytoskeleton component glial fibrillary acidic protein (GFAP) (12); ubiquitin carboxy-terminal hydrolase L1 (UCH-L1) involved in the neuronal production of ubiquitin (12) as well as neurofilament light (NF-L), the smallest component of the axonal cytoskeleton (13). Today, S100B is used locally as an early screening tool in the Scandinavian Guidelines in mild and moderate TBI (14), where low levels in serum have been shown to be able to safely exclude the presence of intracranial injury in mild TBI patients and thus obviate the needs for head computed tomography in these cases. However, it has been suggested that one of the limitations with the protein is the relatively short serum elimination half-life (suggested to be as short as 25 min in patients with no ongoing brain injury) (15). Thus, in patients with mild/moderate TBI without pathophysiological processes to cause a sustained release in S100B, delayed sampling may be falsely reassuring and this is reflected in the guidelines, which suggest a cutoff of 6 h after trauma (16). It is becoming increasingly clear that a specific level in serum is of little importance if in the absence of kinetic considerations.

It is not completely clear how these proteins leave the injured brain and enter the blood. Blood–brain barrier (BBB) disruption (17) or release independent of BBB integrity (18) as well as passage through the newly discovered glymphatic system (19) have been suggested as possible routes. Presumably, these proteins are first released in the brain extracellular space, a component difficult to access for repeated sampling (20), before being transported to the cerebral spinal fluid (CSF) [where a passive diffusion from CSF to blood the first 24 h after injury has been suggested (21)] and/or subsequently into serum where it is most easily sampled. However, there are several factors that may influence this transport and thus the availability in serum, including clearance, redistribution, protein stability, and ongoing release from the damaged brain (22). The protein S100B has been shown to have a 100% renal clearance (23), and may thus be affected in patients with renal insufficiency (15, 24, 25). Studies regarding serum clearance for the other biomarkers are scarce, but as they are larger, it is probable that liver metabolism is involved (26).

Thus, the serum concentrations of these biomarkers over time are the net sum of complex wash-in (“leak” from the injured brain) and washout (clearance and elimination from the blood) processes that are variable over time, together creating a profile with an expected peak time and a decay rate. This “fall” rate after peak gives rise to what is here termed the effective half-life (*t*_1/2_). This process may vary under different conditions and over time in a way that has not yet been properly studied in biomarker research and is distinct from the elimination half-life. Because of this, it will not be possible to present accurate true serum half-lives of these proteins in TBI cohorts. However, composite peak times and biological half-lives can to some extent be grossly estimated from the literature. In this review, we have chosen to focus specifically on these serum trajectories and temporal profiles after TBI.

While there have been several systematic review articles addressing the utility of different biomarkers in detecting injury and predicting outcome (16, 27, 28), there are no studies that have systematically integrated the current knowledge concerning serial sampling of serum biomarkers in brain injured patients, with the goal of suggesting interpretation of levels and estimating peak times and biological half-lives. Understanding the temporal profiles of biomarkers is crucial, as it will provide pertinent information on how to interpret trends. Additionally, current and ongoing studies assessing treatment efficacy (29) as well as multicenter TBI studies such as CENTER-TBI are providing researchers with large cohorts of serial serum samples, where the utility of serial sampling in monitoring secondary events could be assessed (30).

### Aim

By systematically and comprehensively reviewing the available literature on serial serum biomarker sampling in human TBI, we wish to assess temporal trajectories in order to better understand serum *t*_1/2_ of these proteins.

## Materials and Methods

A systematic review was performed, using the methodology outlined in the Cochrane Handbook for Systematic Reviewers (31). Data were reported following the Preferred Reporting Items for Systematic Reviews and Meta-Analyses (PRISMA) (see Data Sheet S1 in Supplementary Material for PRISMA checklist) (32).

### Search Question and Population of Interest

The main question posed for this scoping systematic review was as follows: How do serum S100B, NSE, GFAP, UCH-L1, and NF-L levels change with time following TBI? Thus, we aimed to include all studies reporting at least two serum samples of S100B, NSE, GFAP, UCH-L1, or NF-L (respectively) in human TBI. The primary outcome of interest was serum dynamics, and the resulting effective serum half-life (*t*_1/2_), over time for these proteins.

### Inclusion/Exclusion Criteria

#### Inclusion Criteria

All studies including human subjects with TBI, any study size, any age category, and reporting at least two serum samples of either/or S100B, NSE, GFAP, UCH-L1, and/or NF-L.

#### Exclusion Criteria

Animal studies, non-TBI studies, studies without neuroradiological examinations, studies analyzing the biomarker in other bodily compartments than serum, non-English studies (very few available, only one such study *n* = 1 for S100B was found that could otherwise have been potentially included), meeting abstracts, and studies failing to adequately demonstrate data from multiple sampling.

### Search Strategies

MEDLINE, EMBASE, and SCOPUS from December 1, 1945 to January 31, 2017 were searched using similar search strategies, which were individualized for each database interface. The search strategy using MEDLINE and EMBASE can be seen in Data Sheet S2 in Supplementary Material, with a similar search strategy utilized for the other database. All possible MESH-terms were used for the different biomarkers (Data Sheet S2 in Supplementary Material). Reference lists of any review articles on this subject were reviewed for any missed relevant studies.

### Study Selection

Utilizing two reviewers, a two-step review of all articles returned by our search strategies was performed. First, the reviewers independently (Frederick Adam Zeiler and Eric Peter Thelin) screened titles and abstracts of the returned articles to decide if they met the inclusion criteria. Any meeting abstracts returned by the search strategy were not included in the final review. However, we hand searched the abovementioned databases for any published manuscripts corresponding to these meeting abstracts, prior to discarding them. Second, full texts of the chosen articles were then assessed, to confirm if they met the inclusion criteria and that the primary outcome of interest was reported in the study (Frederick Adam Zeiler and Eric Peter Thelin). Any discrepancies between the two reviewers were resolved by a third reviewer if needed (Adel Helmy or David K. Menon).

### Data Extraction

Using a tailored form, data were extracted from the selected articles and stored in an electronic database. Data fields included the following: number of patients, patient demographics [age and injury severity, usually based on Glasgow Coma Scale (GCS) (33)], type of assay used (technique and manufacturer, if available), sampling frequency, trend over time for the specific biomarker (looking at serum data either in tables or figures presented in the articles), estimated temporal profile/serum availability, and any specific notes concerning the serial sampling of the biomarker. As delta values were not provided, to calculate the *t*_1/2_ the concentration decrease was divided by time. So, if the first concentration following the peak was 0.50 µg/L and a second sample acquired after 12 h was 0.25 µg/L, it would have resulted in a *t*_1/2_ of 12 h. If the serum concentration initially increased, the decrease following the peak concentration was used (and was commented on in “notes”). If the concentration only increased over time, it was commented on, and no *t*_1/2_ was calculated. In the case of long sampling frequency, a non-accurate range for the effective serum half-life was noted.

### Quality/Bias Assessment

As only three S100B studies and one UCH-L1 study (29–32) investigated serum dynamics over time, a formal bias assessment of all the included studies is not possible. However, as the goal of this review was to produce a systematically conducted review of the available literature on serial sampling of serum biomarkers in TBI, this is not critical. Our main aim was instead to produce a comprehensive overview of the current literature on the topic.

### Statistical Analysis

Due to the heterogeneity of the data, both between severity grades of included TBI patients and varying sampling times and assays used, we could not perform a formal meta-analysis of the collected data. However, we did make histograms for the different biomarker studies with a sampling frequency of 24 h or less that indicated an estimate of *t*_1/2_.

## Results

### S100B

**Figure 1:**
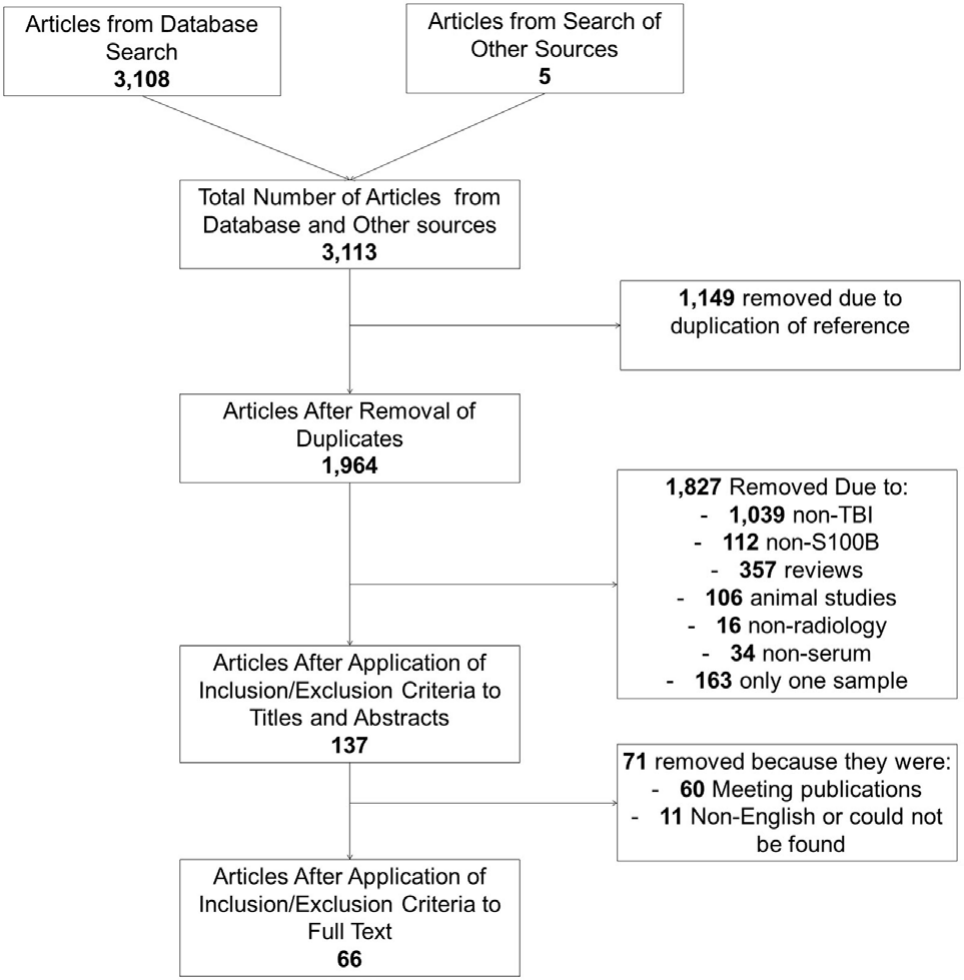
Illustrating the selection of articles with serial sampling of S100B.

A search for S100B identified a total of 3,113 manuscripts. Following removal of duplicates and after assessing full manuscripts, 66 articles were deemed eligible for final inclusion (Figure 1) and are listed in Table 1.

**Table 1:** Analysis of S100B studies.

Reference	Number of patients	Patient characteristics	S100B assay	Sampling frequency	Trend over time	Suggested effective half-life	Notes
Akhtar et al. (62)	17 (7 with TBI)	Pediatric (5–18 years), mild TBI	Liaison, Sangtec	6 h (only two samples)	Decreases first 12 h after trauma	None stated, >6 h	No specific kinetic monitoring. Higher levels in patients with lesions on MRI
Baker et al. (34)	64	Adult, severe TBI patients (GCS < 9)	ELISA, Nanogen Corp.	Initially, 12 h	Decreases first 48 h after trauma, does not reach control levels	None stated, <12 h	No specific kinetic monitoring. Higher levels in patients not treated with hypertonic saline
Berger et al. (67)	100	Pediatric, inflicted and non-inflicted TBI cases. GCS 3–15	ELISA, Nanogen Corp.	12 h	Inflicted TBI longer time-to-peak S100B than non-inflicted TBI	None stated, not enough data to suggest one	Worse GCS have longer time-to-peak
Blyth et al. (68)	10	Adult (39–63 years) mild-to-severe (3–14) TBI	ELISA, Nanogen Corp.	Initially 12 h	S100B levels reach healthy control after 48 h	None stated, 24–48 h	Levels all time below reference levels
Buonora et al. (69)	154 (106 with TBI)	Adult mild-to-severe TBI (GCS 3–15)	TBI 6-Plex, MSD	>48 h	Decreasing quickly over time	None stated, <6 h	No specific outcome concerning biomarker kinetics
Chabok et al. (35)	28	Adult, severe (GCS < 9) diffuse axonal injury TBI	ELISA, CanAg Diagnostics	About 24 h	Decreases quickly over time	None stated, difficult to say, <24 h	Later S100B levels better outcome predictors
Chatfield et al. (70)	20	Adult (16–60 years), moderate-to-severe (GCS 3–11) TBI	LIA-mat, Sangtec	24 h	Decreases over time, reaches control after 5 days	None stated, about 24 h	No specific outcome concerning biomarker kinetics
DeFazio et al. (36)	44	Adult (16–64 years) severe (GCS 3–8) TBI	Unknown	24 h	Decreases the first 72 h	None stated, <24 h	Higher levels in patients with unfavorable outcome
Di Battista et al. (71)	85	Adult moderate-to-severe TBI	Multiplex immunoassay system, MSD	Initially, every 6 h	Quickly declining the first 24 h	None stated, <6 h	Higher levels in patients with unfavorable outcome
Dimopoulou et al. (37)	47	Adult (17–75 years), severe (GCS < 9) TBI	LIA-mat, Sangtec	24 h	Decreases in non-brain dead patients until day 5. Increase in brain dead patients	None stated, 3 days in non-brain dead patients	Higher levels and more volatility in brain dead patients
Elting et al. (72)	10	Adult, moderate-to-severe (GCS 3–13) TBI	LIA-mat, Sangtec	24 h	Decreases the first days, baseline after about 9 days	None stated, about 3 days	No specific outcome concerning biomarker kinetics
Enochsson et al. (63)	19	Adult, mild TBI patients	LIA-mat, Sangtec	4 h (one sample only)	Returns to normal within 4–6 h	4–6 h suggested, looks probable in most patients	No different in kinetics with ethanol in the blood
Ercole et al. (73)	154	Adult, mild-to-severe (GCS 3–15), NICU TBI	CLIA, Liaison, DiaSorin and ECLIA, Elecsys, Roche	12 h	S100B peaks at 27.2 h	None stated, varying over time	Kinetics specifically mapped in patients without secondary peaks of S100B
Ghori et al. (38)	28	Adult (18–65 years), severe (GCS 3–7) TBI	LIA-mat, Sangtec	24 h	Good outcome patients stabilize after 3 days, poor outcome patients increased even after 5 days	None stated, 24 h in patients with good outcome, 72 h in patients with poor outcome	Higher levels in patients with unfavorable outcome
Goyal et al. (21)	80	Adult, severe (GCS < 9) TBI	ELISA, Nanogen Corp.	24 h	Slowly decreasing levels (peak at 24 h), more quickly decrease in patients with good outcome the first 5 days	None stated, about 24 h in patients with favorable outcome and 72 h in patients with unfavorable outcome	Possible to divide patients in trajectory groups where higher levels over time are correlated with an unfavorable outcome
Herrmann et al. (74)	69	Adult (16–67 years) mild-to-severe TBI patients (GCS 3–15)	LIA-mat system, Sangtec	About 24 h	Quickly declining first 12 h, then a plateau until 73 h	None stated, presumably <12 h	Earlier samples better for outcome prediction
Herrmann et al. (75)	66	Adult (16–65 years) mild-to-severe TBI patients (GCS 3–15)	LIA-mat system, Sangtec	24 h	Slowly declining, in some pathologies secondary peaks occurred	None stated, presumably about 24–48 h	Higher in different types of pathologies over time (diffuse axonal injury and edema)
Herrmann et al. (76)	69	Adult (16–65 years) mild-to-severe TBI patients (GCS 3–15)	LIA-mat system, Sangtec	24 h	Relatively slow decline over 96 h	None stated, presumably 49–72 h	Higher area under curve levels in unfavorable outcome. S100B increased 2 weeks and 6 months after injury
Honda et al. (77)	34 (18 TBI patients)	Adult ED TBI patients (GCS 5–14)	ELISA, Yanaihara Institute	24 h	Constantly increased the first 3 days	None stated, presumably >72 h	No specific analysis on biomarker kinetics
Ingebrigtsen et al. (64)	50 (10 patients highlighted)	All ages (6–88 years), mild (GCS 14–15) TBI patients	LIA-mat system, Sangtec	6–12 h (only two samples)	Rapidly decreasing the first 12 h	None stated, <12 h	Early sampled S100B samples decrease rapidly
Ingebrigtsen and Romner (65)	2	All ages (12–73 years), mild (GCS 14–15) TBI patients	LIA-mat system, Sangtec	1 h	Decreasing the first 8 h in patients with injuries on MRI	None stated, about 6 h	Patients with injuries on MRI have elevated S100B levels
Jackson et al. (39)	30	Severe TBI patients	ILA, Byk-Sangtec	3–4 h (only two samples)	Decreasing the first 240 h.	198 min (100 to >500 min presented)	The patients with the highest levels had the most rapid decreases
Joseph et al. (40)	40	Adult (>17 years), severe (GCS < 9) TBI	ELISA, BioVendor	Initially 6, then 18 h	Patients with remote ischemic conditioning decrease over time	None stated, >24 h	No specific analysis on biomarker kinetics
Kellermann et al. (78)	57	Adult, moderate-to-severe TBI	ECLIA, Elecsys, Roche	24 h	Decreasing the first 4–5 days	None stated, about 96 h	No specific analysis on biomarker kinetics. Significant decrease over time
Kleindienst et al. (18)	71	Adult (>17 years), mainly severe TBI	ECLIA, Cobas, Roche	24 h	Steadily decreasing, under reference levels after 20 days	None stated, about 48–72 h	Does not seem to be a kinetic association between CSF and serum
Kleindienst et al. (79)	19	Adult, severe TBI	ECLIA, Elecsys, Roche	24 h	Initial peak to day 2, then decline the first 10 days	None stated, about 96 h	No specific analysis on biomarker kinetics
Korfias et al. (41)	112	Adult (16–86 years), severe (GCS < 9) TBI	LIA-mat system, Sangtec	24 h	Decreasing steadily for survivors, plateau in 96 h. Remaining increased in non-survivors	None stated, about 48 h in survivors and a lot longer in non-survivors	Neurological deterioration during the clinical course is related to increases in S100B
Li et al. (42)	159	Adult (15–71 years) severe (GCS < 9) TBI	ELISA, unknown origin	3 days	Decreases over time, very slow decrease in control group	None stated, 14 days in the treated group, >3 months in the control group	Lower S100B levels over time in the erythropoietin group
McKeating et al. (80)	21	Adult (17–69 years) moderate-to-severe (GCS 3–13) TBI	LIA-mat system, Sangtec	24 h	Decrease over time, up to 48 h, some outliers with increasing levels	None stated, presumably >24–48 h in unaffected patients	More volatility in patients with unfavorable outcome
Mofid et al. (81)	32	Adult, mild-to-moderate TBI	ELISA, BioVendor	24 h and 5 days	Decreasing, and plateauing during 24 h for the progesterone group, while constantly increased for controls	None stated, <24 h in treated patients and >6 days in controls	Lower S100B levels over time in the progesterone group
Murillo-Cabezas et al. (43)	87	Adult (15–76 years), severe (GCS < 9) TBI	ECLIA, Elecsys, Roche	24 h	Decreasing the first 3 days	None stated, 24–48 h	Longer serum half-life in patients with unfavorable outcome
Nirula et al. (82)	16	Adult mild-to-severe TBI	ILA system, Sangtec	24 h	Decrease first 3 days, then stabilizing	None stated, presumably < 24 h	Higher levels in patients with placebo treatment
Nylén et al. (44)	59	All age (8–81 years), severe (GCS < 9) TBI	ELISA, Fujirebio	24 h	Decrease the first 4 days, then plateauing	None stated, 24–48 h	S100BB and S100A1B have slower declines in serum than S100B
Olivecrona et al. (45)	48	Adult (15–63 years), severe (GCS 3–8) TBI	CLIA, Liaison, Sangtec	12 h	Elevated first 4 days, then steep decrease	None stated, presumably about 120 h	Worse correlation between NSE and S100B as time progresses
Olivecrona and Koskinen (46)	48	Adult, severe (GCS < 9) TBI patients	CLIA, Liaison, Sangtec	12 h	Decrease the first 2 days, then stabilizing in ApoE4 groups. Longer time elevated in non-Apo-E4 patients	None stated, about 48 h in Apo-E4 groups, longer in non-Apo-E4 patients	APO-E4 patients have lower S100B levels over time
Olivecrona et al. (47)	48	Adult, severe (GCS < 9) TBI patients	CLIA, Liaison, Sangtec	12 h	Decrease the first three days, then stabilizing	None stated, about 80 h	Later S100B levels better for outcome prediction
Pelinka et al. (83)	79	Adult, mild-to-moderate TBI	LIA-mat system, Sangtec	24 h	Quick decrease for early <12 h samples to 12–36 h. Decrease the first 108 h	None stated, about <12 h	Later S100B levels better for outcome prediction
Pelinka et al. (48)	46	Adult, severe (GCS < 9) TBI	LIA-mat system, Sangtec	24 h	Very high early levels that stabilize after 96 h, especially in multitrauma patients	None stated, 12–24 h	Brain injuries more prolonged release than extracranial trauma
Pelinka et al. (84)	92	Adult, mild-to-severe TBI patients	CLIA, Liaison, Sangtec	24 h	Very high early levels (especially in non-survivors) that stabilize after about 60 h	None stated, 12–24 h	Later S100B levels better for outcome prediction
Petzold et al. (86)	21	Adult, mild-to-severe TBI	ELISA, custom made	24 h	High levels in non-survivors that decrease over time. Little change in survivors that have similar levels as healthy controls after 6 days	None stated, about 72 h for non-survivors	Difficult to compare the levels, are a lot higher than other studies
Petzold et al. (85)	14	Adult (23–56 years), severe TBI	ELISA, custom made	24 h	Slight increase the first day, then a steady decline the first 6 days	None stated, about 6 days	Timing important for S100B interpretation
Piazza et al. (87)	12	Pediatric (1–15 years), mild-to-severe (GCS 3–15) TBI	CLIA, Liaison, Sangtec	48 h (only two samples)	Very heterogeneous trajectories for different patients	None stated, not possible to say	No specific analysis on biomarker kinetics
Pleines et al. (49)	13	Adult (16–67 years), severe TBI (GCS < 9)	ELISA, Sangtec	24 h	Drops relatively quick, “normal” levels after 5 days	None stated, difficult due to log data but probably 48–72 h	No specific analysis on biomarker kinetics
Raabe et al. (50)	15	Adult (19–58 years), severe (GCS < 9)	LIA-mat, Sangtec	24 h	Some patients increase, other steady over time, while many decrease the first 5 days	None stated, difficult to say due to few patients, probably about 48 h in a majority of patients	Patients with secondary increases have a more unfavorable outcome
Raabe and Seifert (51)	3	Adult (17–55 years), severe (GCS < 9) TBI	Unknown	24 h	Secondary increases in three patients	None stated, impossible to say	Secondary increases lead to fatal outcome
Raabe et al. (88)	84	Adult (16–85 years), severe (GCS < 9) TBI	LIA-mat, Sangtec	24 h	Very diverse temporal trajectories in non-surviving patients, steady decline in surviving patients	None stated, difficult to say for non-survivors, probably 24–48 h in survivors	Later samples better for outcome prediction
Raabe and Seifert (89)	25	Adult (18–78 years), severe (GCS < 9) TBI	LIA-mat, Sangtec	24 h	Very dynamic trajectory in patients with unfavorable outcome, steady decline in patients with favorable outcome	None stated, about 72 h in patients with favorable outcome	No specific analysis on biomarker kinetics
Raabe et al. (7)	31	Adult, severe (GCS < 9) TBI patients	CLIA, Liaison, Sangtec	24 h	Increase in TBI patient with cerebral infarction	None stated, difficult to say as only one TBI patient is illustrated	Secondary peaks correlated with a secondary deterioration
Raheja et al. (52)	86	Adult (18–65 years), severe TBI (GCS 4–8)	ELISA, BioVendor	7 days	Decrease the first 7 days	None stated, <7 days	No specific analysis on biomarker kinetics
Rodriguez-Rodriguez et al. (53)	56	Adult, severe TBI (GCS < 9)	ECLIA, Elecsys, Roche	24 h	Steady decline, the first 96 h	None stated, about 24 h	Admission samples worse than 24 h S100B samples for outcome
Rodriguez-Rodriguez et al. (54)	99	Adult, severe TBI (GCS 3–8)	ECLIA, Elecsys, Roche	24 h	Decreasing the first 96 h, greater decrease in patient with better outcome	None stated, presumably 24 h for both survivors and non-survivors	72 h S100B is best for outcome prediction
Rothoerl et al. (55)	15	Adult (17–73 years), severe (GCS < 9) TBI	RIA, Byk-Sangtec	Initially 6, then 24 h	Patients with unfavorable outcome peak at 6 h after admission and then decreasing, favorable outcome patients decrease constantly	None stated, <6 h in patients with favorable outcome and 24 h with unfavorable	No specific analysis on biomarker kinetics
Shahim et al. (56)	72	Adult, severe (GCS < 9) TBI	ECLIA, Cobas, Roche	24 h	Decreases steadily over time (12 days). All normal after 1 year	Not mentioned, 24–48 h	No specific analysis on biomarker kinetics
Shakeri et al. (57)	72	All ages (5–80 years), severe (GCS < 9) TBI	ELISA (?), unknown origin	Initially 48 h	Higher levels in brain dead patients after 48 h than in favorable outcome	None stated, difficult to say due to different sampling.	Highest in patients diagnosed as brain dead
Thelin et al. (11)	417	Adult (>14 years old), mild-to-severe (GCS 3–15) NICU TBI patients	CLIA, Liaison, DiaSorin and Elecsys, Roche	12 h	Decreasing over the first 60 h, faster in patients with favorable outcome. Peaks at about 30 h	None stated, about <6 h initially but longer in later (24 h) samples	S100B influenced by multitrauma first 10 h. 30-h samples best for outcome prediction. More volatility and higher levels in patients with poor outcome
Ucar et al. (58)	48	Severe (GCS < 9) TBI	LIA-mat, Sangtec	48 h	Higher levels on day 3 for the unfavorable group, otherwise unchanged over time	None stated, difficult to suggest one	Patients with unfavorable outcome secondary peaks of S100B
Undén et al. (59)	1	Severe (GCS3) TBI, 22 years old	CLIA, Liaison, Sangtec	Hourly	Very volatile S100B dynamics over time in patient with TBI that succumbs due to cerebral herniation	None stated, difficult to suggest one	Intracranial perfusion necessary for S100B release
Undén et al. (90)	29 TBI	Adult, mild-to-moderate, NICU TBI	CLIA, Liaison, Sangtec	24 h	Elevation > 0.5 μg/L harmful deterioration	None stated, difficult to assess	Strong association between S100B levels and secondary complications
Vajtr et al. (94)	18	Unknown TBI	ECLIA, Elecsys, Roche	>3 days	Decreasing over the first 7–10 days, more so in the less injured group	None stated, probably <3 days.	Decreasing a lot quicker in patients who did not need neurosurgery
Vajtr et al. (95)	38	Different types of presumably adult, severe TBI	ECLIA, Cobas, Roche	>3 days	Decreasing over 1–3 vs 4–10 days in all intracranial pathologies	None stated, <72 h.	Non-expansive contusions highest S100B over time
Walder et al. (91)	49	Severe (AIS > 3, but GCS 3–10), adult TBI	ELISA, Abnova Corp.	Initially 6, then 24 h	Decreases quickly the first 12 h, then more stable	None stated, presumably around 6 h	No difference between multitrauma and non-multitrauma patients. Higher early S100B levels in patients with GCS 3–8
Watt et al. (60)	23	Adult (18–34 years), severe (GCS < 9)	LIA-mat, Sangtec	24 h	Decreases steadily with constant half-life the first 6 days, then leveling	None stated, between 24–48 h	Early samples drawn, quick decline. High early levels associated with an unfavorable outcome
Welch et al. (92)	167	Adult moderate-to-mild TBI (GCS 9–15)	ECLIA, Cobas, Roche	Every 6 h (up to 24 h)	Generally decreasing trends, some increase the first 12 h	None stated, some shorter but seems to <12 h for a majority of patients	After about 8 h, all patients with extracranial injury levels have low levels of S100B
Woertgen et al. (66)	30	Adult (17–73 years), severe (GCS 3–8) TBI	RIA, Byk-Sangtec	Initially 6 h	Decreasing the first hours, then increasing at 24 h with a secondary peak, only to decline later on the first 120 h	None stated, <6 h in early samples but with a secondary increase	Early levels reveal quick early decrease and higher levels in patients with more unfavorable outcome
Yan et al. (61)	42	Adult (16–63 years), severe (GCS < 9) TBI	ELISA, Diasorin	24 h	Steadily decreasing the first 5 days, almost reaching same levels as seen in healthy controls	None stated, 24–48 h	No specific analysis on biomarker kinetics
Zurek and Fedora (93)	63	Pediatric (0–18 years), presumably different severity of injury	ECLIA, Elecsys, Roche	24 h	Steadily declining the first 5 days, some outliers with higher levels	None stated, <24 h for a majority of patients. Some have secondary peaks	Early levels reveal quick early decrease and higher levels in patients with more unfavorable outcome

*Number of patients highlighted the total number of patients included in the study, sometimes highlighting in parenthesis how many were actually included with TBI or serial sampling. Patient characteristics described the age groups and injury severity level according to the GCS. The assay described the technique used for the assay and if available the manufacturer. Sampling frequency illustrates with what frequency samples were acquired. Trewnd over time highlights the specific temporal trajectory and dynamics for S100B. Suggested effective serum half-life is noted, as derived from graphs or tables. “Notes” indicate any specific considerations or notable findings of serial sampling in the specific article*.

*TBI, traumatic brain injury; ECLIA, electrochemiluminescent immunoassay; ELISA, enzyme-linked immunosorbent assay; GCS, Glasgow Coma Scale; ED, emergency department; ILA, immunoluminometric assay; RIA, radioimmunoassay; CLIA, chemiluminescent immunoassays; ECLIA, electrochemiluminescent immunoassays; NICU, neurointensive care unit; NSE, neuron-specific enolase; CSF, cerebral spinal fluid*.

#### Patient Characteristics

Generally, the patient characteristics in the S100B studies were severely injured TBI patients (GCS < 9 at admission, unconscious) (21, 34–61) and mild (GCS 14–15) (62–66) or in combination (including moderate GCS 9–13) (7, 11, 18, 67–92) (Table 1). All different age groups were analyzed, including only or partial pediatric populations (44, 57, 62, 64, 65, 67, 93), even if a vast majority included solely adult patients (7, 11, 18, 21, 34–38, 40–43, 45–51, 53–56, 59–61, 63, 66, 68, 71–92).

#### Assays Used to Analyze Serum S100B

The studies used a wide variety of different assays to analyze S100B. Commercial or custom made enzyme-linked immunosorbent assays (ELISAs) were used in some studies (21, 34, 35, 40, 42, 44, 49, 52, 57, 61, 67, 68, 77, 81, 85, 86, 91), as well as other techniques (or not mentioned in the text) (36, 51, 55, 66, 69, 71), even if a majority used clinically available assays such as the (C)LIA-mat system from Sangtec/DiaSorin (7, 11, 18, 37–39, 41, 43, 45–48, 50, 53, 54, 56, 58–60, 62–65, 70, 72–76, 78–80, 82–84, 87–90, 92–95). In general, ELISA samples showed less volatility over time (42, 49, 68, 81, 91). Specifically, they tended to have elevated levels over a prolonged period of time as compared to the automated, clinical assays (42, 49, 52, 77, 81, 85, 86, 91).

#### Sampling Frequency of S100B

While some studies had more than 24 h between sampling times (42, 52, 57, 58, 81, 87, 94, 95), generally the studies had a sampling frequency of every 24 h (7, 18, 21, 35–38, 41, 43, 44, 48–51, 53, 54, 56, 60, 61, 70, 72, 74–86, 88–90, 93), or sometimes twice daily (11, 34, 45–47, 67, 68, 73), or every 4–6 h (39, 40, 55, 62–64, 66, 69, 71, 91, 92). In contrast to the other biomarkers, there were some studies that assessed S100B hourly in order to track the serum dynamics (59, 65).

#### Trend of S100B over Time after Trauma

Following trauma, almost all articles proclaim a steadily decline in levels of S100B (18, 21, 35, 36, 40, 43–48, 52–54, 56, 60, 61, 64, 69–72, 78, 80–84, 86, 88, 91, 93–95), while some suggested a slight increase before declining (55, 73, 79, 85, 92). Patients suffering from multitrauma and TBI were seen to have higher levels compared to patients with only TBI (11, 48, 92). Generally, the decreasing trajectory of S100B was strongly correlated with the severity of trauma and/or the outcome for the patient (11, 21, 36–38, 41–43, 50, 54, 55, 58, 60, 62, 65, 66, 71, 75, 84, 86, 93–95). Some papers indicate a very quick decline of serum S100B (39, 49, 83), while some suggest that it is a slower decline over time in relation to when the sample is acquired (11, 60, 64, 69, 71, 73, 74, 85, 93). Several studies indicate volatile S100B trajectories in more unstable patients with detrimental outcomes due to injury development (37, 38, 41, 50, 57, 59, 66, 67, 80, 84, 87–89). Secondary increases (“peaks”) of S100B were found in several studies and correlated with secondary adverse events (7, 41, 51, 58, 75, 90). Some clinical trials noted a faster decrease of S100B in serum over time in the trial group as compared to placebo (34, 42, 81, 82).

#### Suggested Serum *t*_1/2_ of S100B

**Figure 2:**
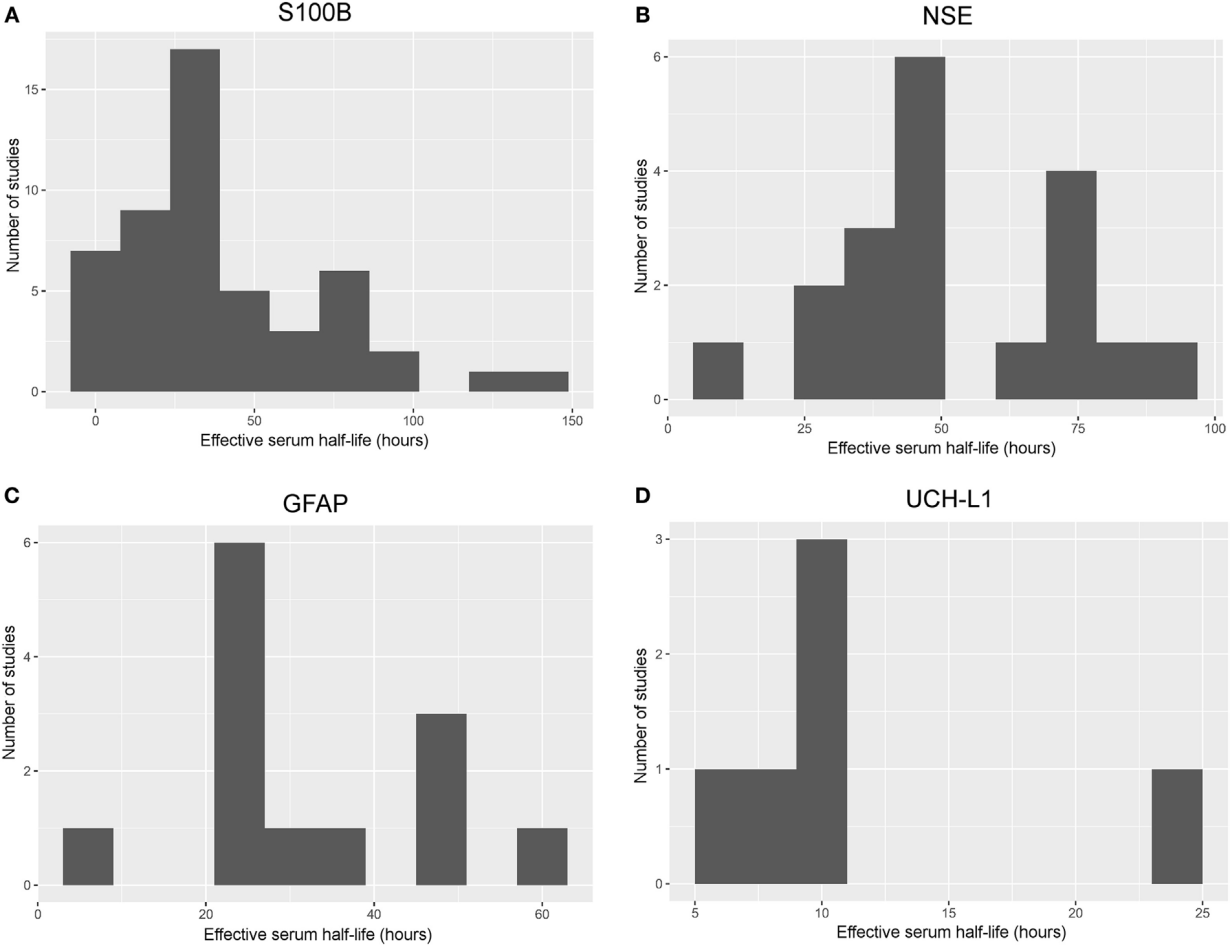
Histograms of frequency of effective serum half-life in different studies. Histograms illustrating the aggregated effective serum half-lives as derived from the different studies including S100B **(A)**, neuron-specific enolase (NSE) **(B)**, glial fibrillary acidic protein (GFAP) **(C)**, and ubiquitin carboxy-terminal hydrolase L1 (UCH-L1) **(D)**. Studies with a sampling frequency of 24 h or shorter and a valid estimate of the effective serum half-life were included. The bin size is set to 10 h in order to easily visualize trends; a relatively short effective serum half-life for S100B and UCH-L1, while it was longer for NSE and GFAP. An effective serum half-life for neurofilament light could not be included as it was impossible to estimate from the available literature.

Available data suggest that there is a rapid influx and fast *t*_1/2_ of about 2–6 h in mild TBI patients, which is similar in more severe TBI patients if acquired early (11, 34, 39, 55, 63–66, 69, 71, 74, 83, 91, 92) (Figure 2A). Additionally, a slower influx with a later peak and *t*_1/2_ of about 24 h the first days in severe TBI patients are described (11, 21, 35, 36, 38, 40, 43, 44, 48, 53, 54, 56, 60, 61, 70, 73, 75, 81, 82, 84, 88, 93). Some studies reported *t*_1/2_ of days (24–120 h) (18, 21, 37, 41, 45–47, 49, 72, 76–80, 85, 86, 89) or even weeks (14 days) (42).

### Neuron-Specific Enolase

A search for NSE identified a total of 4,511 manuscripts. Following the removal of duplicates and after assessing full manuscripts, 27 articles were deemed eligible for final inclusion (Figure S1 in Supplementary Material) and are listed in Table 2.

**Table 2:** Analysis of NSE studies.

Reference	Number of patients	Patient characteristics	NSE assay	Sampling frequency	Trend over time	Suggested reactive half-life	Notes
Baker et al. (34)	70	Adult, severe TBI patients (GCS < 9)	ELISA, Nanogen Corp.	Initially, 12 h	Decreases quickly after trauma	None stated, 12–15 h the first hours	No specific kinetic monitoring. Higher levels in patients not treated with hypertonic saline
Beers et al. (102)	30	Pediatric TBI (GCS 3–15)	ELISA, Nanogen Corp.	12 h	Increases the first 4 days in inflicted trauma	None stated, not enough data to suggest one	Worse outcome if longer time to peak levels
Berger et al. (67)	100	Pediatric, inflicted, and non-inflicted TBI cases. GCS 3–15	ELISA, Nanogen Corp.	12 h	Inflicted TBI longer time-to-peak NSE than non-inflicted TBI	None stated, not enough data to suggest one	Patients with lower GCS have longer time-to-peak
Buonora et al. (69)	154 (106 with TBI)	Adult mild-to-severe TBI (GCS 3–15)	TBI 6-Plex, MSD	>48 h	Decreasing steadily over time	None stated, about 24 h	No specific outcome concerning biomarker kinetics
Dauberschmidt et al. (99)	9	Severe TBI patients (GCS 4)	RIA	24 h	Steadily increasing in some, unchanged in some, over 10 days	None stated, not enough data to suggest one	No specific outcome concerning biomarker kinetics
Di Battista et al. (71)	85	Adult moderate-to-severe TBI	Multiplex immunoassay system, MSD	Initially, every 6 h	Slowly declining the first 24 h	None stated, probably >24 h (closer to 48 h)	Primary: First 24 h kinetics studied. No difference in NSE levels between outcome
Guzel et al. (96)	169	Mild-to-severe TBI patients	ECLIA, Cobas, Roche	24 h	Declining over time	None stated, presumably close to 48 h for the entire cohort	Slower decline in patients with more severe injuries
Herrmann et al. (74)	69	Adult (16–67 years) mild-to-severe TBI patients (GCS 3–15)	LIA-mat system, Sangtec	About 24 h	Declining over time, stabilizing after 73 h	None stated, presumably 48 h	Later samples not better for outcome prediction
Herrmann et al. (75)	66	Adult (16–65 years) mild-to-severe TBI patients (GCS 3–15)	LIA-mat system, Sangtec	24 h	Slowly declining, in some pathologies secondary peaks occurred	None stated, presumably 73–96 h	Higher in different types of pathologies over time (diffuse axonal injury and edema)
Herrmann et al. (76)	69	Adult (16–65 years) mild-to-severe TBI patients (GCS 3–15)	LIA-mat system, Sangtec	24 h	Slowly declining over 96 h	None stated, presumably 49–72 h	No association between prolonged increases (6 months) of NSE and outcome
Honda et al. (77)	34 (18 TBI patients)	Adult ED TBI patients (GCS 5–14)	ELISA, Alpha Diagnostics	24 h	Constantly increased the first 3 days	None stated, presumably >72 h	No specific analysis on biomarker kinetics
Li et al. (42)	159	Adult (15–71 years) severe (GCS < 9) TBI	ELISA, unknown origin	Initially, 3 days	Decreases over time, very slow decrease in control group not exposed to erythropoietin	None stated, >14 days in the control, 10–14 days in the treated group	Lower NSE levels over time in the erythropoietin group
McKeating et al. (80)	21	Adult (17–69 years) moderate-to-severe (GCS 3–13) TBI	LIA-mat system, Sangtec	24 h	Decrease over time, up to 96 h	None stated, presumably >96 h	More volatility in patients with unfavorable outcome
Nirula et al. (82)	16	Adult mild-to-severe TBI	ILA system, Sangtec	24 h	Decrease first 3 days, then stabilizing	None stated, presumably about 48 h	Higher levels in patients with erythropoietin treatment
Olivecrona et al. (45)	48	Adult (15–63 years), severe (GCS 3–8) TBI	CLIA, Liaison, Sangtec	12 h	Decrease the first 3 days, then stabilizing	None stated, presumably about 72 h	Worse correlation between NSE and S100B as time after trauma increases
Olivecrona and Koskinen (46)	48	Adult, severe (GCS < 9) TBI patients	CLIA, Liaison, Sangtec	12 h	Decrease the first 3 days, then stabilizing	None stated, presumably about 72 h	APO-E4 patients lower NSE levels over time
Olivecrona et al. (47)	48	Adult, severe (GCS < 9) TBI patients	CLIA, Liaison, Sangtec	12 h	Decrease the first 3 days, then stabilizing	30 h is stated in discussion (no reference), but looks more like 72 h	Later NSE levels better for outcome prediction
Pleines et al. (49)	13	Adult (16–67 years), severe TBI (GCS < 9)	ELISA, Sangtec	24 h	Largely unchanged the first 14 days, slight decrease first day only	None stated, not possible to suggest based on the data	NSE not above reference levels
Raheja et al. (52)	86	Adult (18–65 years), severe TBI (GCS 4–8)	ELISA, DRG International	7 days	Decrease the first 7 days	None stated, <7 days	NSE failed to show any significance to injury over time
Rodriguez-Rodriguez et al. (54)	99	Adult, severe TBI (GCS 3–8)	ECLIA, Elecsys, Roche	24 h	Decreasing the first 96 h, faster decrease with better outcome	None stated, presumably survivors about 24 h and non-survivors about 72 h	48 h NSE is best for outcome prediction
Ross et al. (100)	51 (9 with serial sampling)	Adult, severe TBI	RIA, custom made	Varying frequency (<24 h)	Generally constantly decreasing, one increasing	None stated, probably around 24–48 h, shorter for some	Large spread, some patients have normal NSE levels without any good reason
Shahrokhi et al. (97)	32	Adult (18–60 years), male moderate-to-severe TBI (GCS 3–12)	ELISA, unknown origin	24 h to 6 days	Few samples, decreases over time	None stated, <6 days	No specific analysis on biomarker kinetics
Skogseid et al. (98)	60 (42 mild TBI)	Adult, mild-to-severe TBI	RIA, custom made	Varying frequency, hours (<7 h)	Decreasing the first 12 h in a majority of patients, some steadily low, some increasing	None stated, difficult to assess	Extracranial injury lead to increased levels of NSE
Thelin et al. (11)	417	Adult (>14 years old), mild-to-severe (GCS 3–15) NICU TBI patients	CLIA, Liaison, DiaSorin	12 h	Decreasing over the first 60 h, faster in patients with favorable outcome	None stated, about 24–48 h, longer in patients that died	NSE influenced by multitrauma over time. No specific time frame perfect for outcome prediction. More volatility and higher levels in patients with poor outcome
Vajtr et al. (94)	18	Unknown TBI	ECLIA, Elecsys, Roche	>3 days	Decreasing over the first 7–10 days	None stated, probably 7–10 days	Decreasing quicker in patients who did not need neurosurgery
Woertgen et al. (66)	30	Adult (17–73 years), severe (GCS 3–8) TBI	ELISA, Wallac (maybe with RIA from Sangtec)	Initially 6 h	Decreasing steadily to 24 h, then fluctuating	None stated, 24–48 h	Increasing levels of NSE in patients with high intracranial pressure
Yan et al. (61)	42	Adult (16–63 years), severe (GCS < 9) TBI	ELISA, CanAg Diagnostics	24 h	Steadily decreasing the first 5 days to control levels	None stated, <24 h	No specific analysis on biomarker kinetics
Zhao et al. (101)	128	Adult (16–72 years), severe (GCS < 9) TBI patients with diffuse axonal injury	Unknown	>3 days	Decreasing in the group (magnesium sulfate therapy), while it did not in the placebo group up to 7 days	None stated, > 7 days and even longer in the placebo group	Higher NSE levels in the placebo group
Zurek and Fedora (93)	63	Pediatric (0–18 years), presumably different severity of injury	ECLIA, Elecsys, Roche	24 h	Steadily declining the first 5 days, some outliers with higher levels	None stated, <48 h for a majority of patients. Some have secondary peaks	Higher levels in patients with more unfavorable outcome

*Number of patients highlighted the total number of patients included in the study, sometimes highlighting in parenthesis how many were actually included with TBI or serial sampling. Patient characteristics described the age groups and injury severity level according to the GCS. The assay described the technique used for the assay and if available the manufacturer. Sampling frequency illustrates with what frequency samples were acquired. Trend over time highlights the specific temporal trajectory and dynamics for NSE. Suggested effective serum half-life is noted, as derived from graphs or tables. “Notes” indicate any specific considerations or notable findings of serial sampling in the specific article*.

*TBI, traumatic brain injury; ECLIA, electrochemiluminescent immunoassay; ELISA, enzyme-linked immunosorbent; GCS, Glasgow Coma Scale; ED, emergency department; ILA, Immunoluminometric assay; RIA, radioimmunoassay; CLIA, chemiluminescent immunoassays; ECLIA, electrochemiluminescent immunoassays; NSE, neuron-specific enolase*.

#### Patient Characteristics

Generally, the patient characteristics in the NSE studies were very similar to the S100B studies with a variety of primarily adult, mild/moderate-to-severely (11, 71, 74–77, 80, 82, 96–98), or only severely injured patients (34, 42, 45–47, 49, 52, 54, 61, 66, 99–101) (Table 2). However, some looked at more minor injuries and included pe diatric patients (67, 93, 102).

#### Assays Used to Analyze Serum NSE

Similar to S100B, the studies used to analyze NSE utilize a wide variety of different assays. A majority of studies used clinically available assays such as the LIA-mat system from Sangtec/DiaSorin (11, 45–47, 74–76, 80, 82) or Elecsys/Cobas systems from Roche (54, 93, 94, 96), but commercial/custom made ELISAs (34, 42, 49, 52, 61, 66, 67, 77, 97, 102) and other techniques (69, 71, 98–101) were also used. Comparable to results of the S100B, the ELISA methods generally showed higher levels over time and with less dynamics, as compared to the automated assays (42, 49, 52, 97).

#### Sampling Frequency of NSE

Generally, NSE was sampled either every 6 h (66, 71, 98), 12 h (11, 34, 45–47, 67, 102), and 24 h (54, 61, 74–77, 80, 82, 93, 96, 99, 100) in a majority of studies, while some reported longer sampling frequencies (42, 52, 69, 94, 97, 101).

#### Trend of NSE over Time after Trauma

Neuron-specific enolase has not been as extensively analyzed as S100B, but it shows similar characteristics with early high levels that decrease over time (34, 61, 69, 94, 97, 98, 100). However, the levels do not seem to decline with the same velocity as S100B (11, 42, 45–47, 49, 52, 69, 71, 74, 76, 80, 82) and even increase without any known association with outcome/injury in a few cases (77, 98, 99, 102). Nevertheless, a slower decline of NSE is seen in patient with more severe injuries or a more unfavorable outcome in many studies (11, 54, 66, 67, 93, 94, 96), and some increasing trajectories in patients with poor outcome were reported (66, 80, 93). Patients with concomitant extracranial injuries had higher levels of NSE (11, 98). Similar to S100B, secondary peaks of NSE were shown in some studies for patients with progressing injuries (75, 93). Likewise, some clinical trials noted a faster decrease of NSE in serum over time in the trial group as compared to placebo (34, 42, 101).

#### Suggested Serum *t*_1/2_ of NSE

Available data suggest that the serum *t*_1/2_ for NSE is longer than for S100B, presumably around 48–72 h in patients with severe TBI (11, 45–47, 54, 66, 71, 74, 76, 82, 93, 96) or even longer (42, 75, 77, 80, 94) (Figure 2B). However, some studies reported a shorter *t*_1/2_ at 12 (34) or 24 h (61, 69, 100).

### Glial Fibrillary Acidic Protein

A search for GFAP identified a total of 1,953 manuscripts. Following removal of duplicates and after assessing full manuscripts, 18 articles were deemed eligible for final inclusion (Figure S2 in Supplementary Material) and are listed in Table 3.

**Table 3:** Analysis of GFAP studies.

Reference	Number of patients	Patient characteristics	GFAP assay	Sampling frequency	Trend over time	Suggested effective half-life	Notes
Bogoslovsky et al. (103)	34	Adult, 21 mild + 13 moderate-to-severe TBI	Digital array, Quanterix	30–60 days	Measured long after trauma, normalized in 30 days	None stated, <30 days (in all patients)	Long-term biokinetics studied. Same GFAP levels as in healthy controls after 30 days
Di Battista et al. (71)	85	Adult moderate-to-severe TBI	Multiplex immunoassay system, MSD	Initially, every 6 h.	Quickly declining GFAP, levels. Staying low after 6 h	None stated, <6 h	First 24 h kinetics studied. Higher GFAP in patients with unfavorable outcome
Fraser et al. (113)	27	Pediatric severe TBI (GCS < 9)	ELISA, R-Biopharm	24 h	Steadily declining. Normalizing on day 10	None stated, 24 h the first days after injury.	First 10 days biokinetics, no monitoring. Higher GFAP in patients with unfavorable outcome
Honda et al. (77)	34 (18 TBI patients)	Adult ED TBI patients (GCS 5–14)	ELISA, BioVendor	24 h	Steadily declining first 3 days	None stated, 48–72 h	No GFAP level difference between diffuse and focal injury
Kou et al. (111)	9	Adult, mild TBI patients	ECLIA, MSD	6 h (up to 24 h)	Decline and increase in two patients	N/A	Worse dynamics in patient with worse white matter injury
Lei et al. (104)	67	Severe TBI patients (GCS 3–8)	ELISA, BioVendor	24 h	Steadily declining first 3 days, then normalizing	None stated, about 48 h	More volatile dynamics in patients with unfavorable outcome
Lumpkins et al. (105)	51 (39 with TBI)	Adult TBI patients	ELISA, BioVendor	24 h	Decreasing, but only samples on day 1 and day 2	<48 h	GFAP levels second day better for outcome prediction. No monitoring aspect
Missler et al. (106)	25	Adult severe TBI (GCS < 7)	ELISA, custom made	24 h	Increasing the first 24 h	None, only increasing, all patients died within 24 h	Plasma and serum levels similar. Suggesting a very short half-life, shorter than for S100B
Mondello et al. (12)	81	Adult (including five pediatric) severe TBI patients, GCS 3–8	ELISA, BioVendor	6 h	Remaining elevated first 24 h after injury	None stated, >24 h	Higher GFAP, with more volatile dynamic, in mass lesions vs diffuse injury
Nylén et al. (107)	59	Adult, severe TBI patients	ELISA, custom made	24 h	Peak after 24 h, decline until 144 h (below reference)	None stated, probably around 24 h	Outcome prediction better for later samples
Papa et al. (112)	325 (35 TBI patients with injuries)	Adult mild-to-moderate TBI (GCS 9–15)	ELISA, Banyan Biomarkers	Initially, every 4 h	Peak after 16 h, decline until 132 h	None stated, probably around 32 h	More volatile dynamics in patients with injuries and requiring intervention
Pelinka et al. (84)	92	Adult mild-to-severe TBI patients	ILA, LIAISON, Sangtec	24 h	Decreasing steadily in non-survivors, peaking 12–36 h after trauma in survivors	None stated, 61–84 h in non-survivors and around 24–48 h in survivors	Later samples better outcome predictor
Pelinka et al. (108)	114	Adult mild-to-severe TBI patients	ILA, LIAISON, DiaSorin	24 h	Similar to Pelinka et al. (84)	Similar to Pelinka et al. (84)	Similar to Pelinka et al. (84)
Posti et al. (109)	324 (71 patients with injury)	Adult mild-to-severe TBI patients	Randox Biochip, Randox Laboratories	Initially, every 24 h	Moderate-to-severe TBI decreasing while mild TBI steady	None stated, moderate-to-severe TBI about 24 h	Early samples best for outcome prediction
Raheja et al. (52)	86	Adult (18–65 years), severe TBI (GCS 4–8)	ELISA, BioVendor	7 days	Patients with favorable outcome decreasing, unfavorable constant the first 7 days	None stated, <7 days probably	Day 7 samples of GFAP had good precision for outcome prediction
Takala et al. (110)	See Posti et al. (109)	See Posti et al. (109)	See Posti et al. (109)	Initially, every 24 h	See Posti et al. (109)	See Posti et al. (109)	See Posti et al. (109)
Vajtr et al. (95)	38	Adult, severe TBI patients	Biotrak Activity Assay System	>3 days	Decrease from 1–3 to 4–10 days	None stated, <10 days	No specific findings related to dynamics, expansive contusions highest levels of GFAP
Welch et al. (92)	167 (33 patients with injuries)	Adult mild-to-moderate TBI (GCS 9–15)	ELISA, Banyan Biomarkers	Every 6 h (up to 24 h later)	Only increasing the first 24 h	None stated, probably >24 h	Serum concentrations of GFAP less influenced by temporal changes than other biomarkers
Zurek and Fedora (114)	59	Pediatric (0–19 years) severe TBI (GCS < 9).	ELISA, BioVendor	24 h	Generally decreasing the first 3 days, some outliers with dynamic concentrations over time	None stated, probably 24–48 h	Higher levels over time resulted in a general worse outcome

*Number of patients highlighted the total number of patients included in the study, sometimes highlighting in parenthesis how many were actually included with TBI or serial sampling. Patient characteristics described the age groups and injury severity level according to the GCS. The assay described the technique used for the assay and if available the manufacturer. Sampling frequency illustrates with what frequency samples were acquired. Trend over time highlights the specific temporal trajectory and dynamics for GFAP. Suggested effective serum half-life is noted, as derived from graphs or tables. “Notes” indicate any specific considerations or notable findings of serial sampling in the specific article*.

*TBI, traumatic brain injury; ECLIA, electrochemiluminescent immunoassay; ELISA, enzyme-linked immunosorbent assay; GCS, Glasgow Coma Scale; ED, emergency department; ILA, immunoluminometric assay; GFAP, glial fibrillary acidic protein*.

#### Patient Characteristics

Similar to studies analyzing S100B and NSE, the patient characteristics of the GFAP patients were mixed, but with a preponderance toward more severely injured patients (12, 52, 71, 77, 84, 95, 103–110), even if milder cohorts also have been analyzed (92, 111, 112). Some studies looked partly, or solely, at pediatric cohorts (12, 113, 114).

#### Assays Used to Analyze Serum GFAP

A majority of the GFAP studies used various ELISA assays (12, 52, 77, 92, 104–107, 112–114), except two which used an ILA from Liaison™ (84, 108), two studies which used the Randox Biochip™ (109, 110), one an assay from Biotrak™ (95), one used a digital array from Quanterix™ (103), and two that used an immunoassay from MSD™ (71, 111). Currently, there are no clinically available assays. However, fully automated assays are under development.

#### Sampling Frequency of GFAP

Generally, GFAP was sampled every 24 h (77, 84, 104–110, 113, 114) in a majority of studies (one outlier with 30 days between samples (103)), while some had as short as 6 h sampling (12, 71, 92, 111), and one even 4 h initially (112). Two studies had longer sampling frequencies (52, 95).

#### Trend of GFAP over Time after Trauma

Similar to the previously studied markers, GFAP seems to decrease after trauma over time (71, 77, 95, 104, 105, 109, 110, 113). However, some studies noted initially increasing levels, up to about 16–24 h following injury (84, 107, 108, 112). GFAP usually remained elevated for a prolonged period of time, as compared with S100B (12, 84). One study showed limited contribution of extracranial trauma (108). As with the other biomarkers, some studies noted prolonged elevated levels, or even continually increasing levels/volatile dynamics, in patients with unfavorable outcome or worse injuries (52, 71, 84, 104, 106–114).

#### Suggested Serum *t*_1/2_ for GFAP

The *t*_1/2_ for GFAP appears longer than for S100B, most studies reported a *t*_1/2_ at around 24–48 h in severe TBI patients (12, 77, 84, 92, 104, 105, 107–110, 112–114), while some published data suggesting a shorter *t*_1/2_ (71, 106) (Figure 2C).

### Ubiquitin Carboxy-Terminal Hydrolase L1

A search for UCH-L1 identified a total of 234 manuscripts. Following removal of duplicates and after assessing full manuscripts, nine articles were deemed eligible for final inclusion (Figure S3 in Supplementary Material) and are listed in Table 4.

**Table 4:** Analysis of UCH-L1 studies.

Reference	Number of patients	Patient characteristics	UCH-L1 Assay	Sampling frequency	Trend over time	Suggested effective half-life	Notes
Blyth et al. (117)	16	Adult ED TBI patients (GCS 3–15)	ELISA, custom made	Every 12 h	Constantly decreasing on group level	None stated, probably about 10 h	Blood–brain barrier assessment with biomarker measurements over time
Brophy et al. (115)	86	Adult severe TBI (GCS 3–8)	ELISA, custom made	Every 6 h	Constantly decreasing on group level	7–9 h	Longer half-life in patients with more severe injury and worse outcome
Kou et al. (111)	9	Adult mild TBI patients	ECLIA, Banyan Biomarkers	Every 6 h (up to 24 h later)	Slight increase in a patient with brain hemorrhage	N/A	GFAP and UCH-L1 are correlated with extent of white matter injury
Mondello et al. (12)	81	Adult severe TBI patients (GCS 3–8)	ELISA, custom made	Every 6 h	Constantly decreasing	None stated, probably about 10–12 h	Focal injuries faster decrease of UCH-L1
Mondello et al. (116)	95	Adult severe TBI patients (GCS 3–8)	ELISA, custom made	Every 6 h	Constantly decreasing on group level, early falls first 12 h	None stated, probably about 10 h	Earlier UCH-L1 levels better for outcome prediction
Papa et al. (112)	325 (35 TBI patients with injuries)	Adult mild-to-moderate TBI (GCS 9–15)	ELISA, Banyan Biomarkers	Initially, every 4 h	Constantly decreasing on group level	None stated, probably 5–7 h first 24 h. Normalized in about 48 h	Slower decrease of UCH-L1 concentrations in patients with hemorrhage and need for intervention
Posti et al. (109)	324 (71 patients with injury)	Adult mild-to-moderate TBI (GCS 3–15)	Randox Biochip, Randox Laboratories	Initially, every 24 h	In severe-to-moderate TBI, decreasing first 3 days, constant in mild TBI	None, difficult to assess from study, <24 h	Earlier samples better accuracy for injury severity than later samples
Takala et al. (110)	See Posti et al. (109)	See Posti et al. (109)	See Posti et al. (109)	Initially, every 24 h	See Posti et al. (109)	See Posti et al. (109)	See Posti et al. (109)
Welch et al. (92)	167 (33 patients with injuries)	Adult mild-to-moderate TBI (GCS 9–15)	ELISA, Banyan Biomarkers	Every 6 h (up to 24 h later)	Serum concentrations in patients with brain injury constant first 12 h, then decreasing	None, many outliers with constant or increasing levels. A peak is seen at 8 h	No specific kinetic analysis other than faster decreasing in non-TBI patients

*Number of patients highlighted the total number of patients included in the study, sometimes highlighting in parenthesis how many were actually included with TBI or serial sampling. Patient characteristics described the age groups and injury severity level according to the GCS. The assay described the technique used for the assay and if available the manufacturer. Sampling frequency illustrates with what frequency samples were acquired. Trend over time highlights the specific temporal trajectory and dynamics for UCH-L1 Suggested effective serum half-life is noted, as derived from graphs or tables. “Notes” indicate any specific considerations or notable findings of serial sampling in the specific article*.

*TBI, traumatic brain injury; ECLIA, electrochemiluminescent immunoassay; ELISA, enzyme-linked immunosorbent assay; GCS, Glasgow Coma Scale; ED, emergency department; GFAP, glial fibrillary acidic protein; UCH-L1, ubiquitin carboxy-terminal hydrolase L1*.

#### Patient Characteristics

Generally, the patient characteristics in the UCH-L1 studies were somewhat trichotomized with some of the articles focusing more on the milder TBI spectrum (111, 112), while the others included primarily severe (12, 115–117), or a mix of TBI patients (92, 109, 110) (Table 4). No pediatric TBI population was found.

#### Assays Used to Analyze Serum UCH-L1

Currently, no clinically available assays exist to analyze UCH-L1 and all studies used different ELISAs, either custom made (12, 115–117) or commercially available (92, 112) except for two studies which used a Randox Biochip™ method (109, 110) and one with a ECLIA method from Banyan Biomarkers™ (111).

#### Sampling Frequency of UCH-L1

In comparison to the other proteins, most UCH-L1 studies had a 4–6 h (12, 92, 111, 112, 115, 116), or 12 h (117), sampling frequency, allowing for a good estimate of the temporal profile. Two studies had a longer and varying sampling frequency (109, 110).

#### Trend of UCH-L1 over Time after Trauma

In unison with the other markers, UCH-L1 usually decreased steadily following TBI (12, 109, 110, 112, 116, 117). Secondary peaks, or increasing trajectories in patients with serious injuries, were found in patients in a few papers (92, 111, 112, 115, 116). One study suggested that UCH-L1 peaks at around 8 h after injury (92).

#### Suggested Serum *t*_1/2_ of UCH-L1

Looking at the available data, the serum *t*_1/2_ seems to be about 10 h (12, 112, 116, 117) in severe TBI patients, a few hours shorter in milder cases (112) (Figure 2D). In comparison to the other markers, UCH-L1 actually had one study with the goal of establishing a “half-life” of UCH-L1, which was given at 7–9 h (115) in severe TBI patients. This was found shorter in milder TBI cohorts, where data indicated that it was around 6 h (115).

### Neurofilament Light

A search for NF-L identified a total of 575 manuscripts. Following removal of duplicates and after assessing full manuscripts, only *n* = 2 articles were deemed eligible for final inclusion (Figure S4 in Supplementary Material) and are listed in Table 5.

**Table 5:** Analysis of NF-L studies.

Reference	Number of patients	Patient characteristics	NF-L assay	Sampling frequency	Trend over time	Suggested effective half-life	Notes
Al Nimer et al. (13)	182	Adult NICU TBI patients	ELISA, Uman Diagnostics	Varying frequency first 2 weeks	Constantly increasing, unchanged over first week	N/A	No special monitoring aims
Shahim et al. (56)	72	Adult TBI patients, GCS 3–8	Simoa, Quanterix	Initially, every 24 h	Constantly increasing, group level	N/A	No special monitoring aims

*Number of patients highlighted the total number of patients included in the study, sometimes highlighting in parenthesis how many were actually included with TBI or serial sampling. Patient characteristics described the age groups and injury severity level according to the GCS. The assay described the technique used for the assay and if available the manufacturer. Sampling frequency illustrates with what frequency samples were acquired. Trend over time highlights the specific temporal trajectory and dynamics for NF-L. Suggested effective serum half-life is noted, as derived from graphs or tables. “Notes” indicate any specific considerations or notable findings of serial sampling in the specific article*.

*TBI, traumatic brain injury; NICU, neurointensive care unit; ELISA, enzyme-linked immunosorbent assay; Simoa, single molecule array; N/A, not available; NF-L, neurofilament light; GCS, Glasgow Coma Scale*.

#### Patient Characteristics

Only two studies were included which both presented NICU TBI materials with mixed TBI severity according to GCS admission (Table 5) (13, 56). No pediatric TBI population has been studied.

#### Assays to Analyze NF-L

Officially, there are currently no available ELISAs for NF-L in serum. One of the included studies instead used an ELISA assay developed for CSF samples (13), and the other used the newly developed single molecule array technique to create a functional assay (56).

#### Trend of NF-L over Time after Trauma

In contrast to the other serum biomarkers, the two available studies suggest that NF-L levels in serum in a mild-to-severe (and one severe) TBI cohort of NICU patients tend to increase over time during the first 1–2 weeks (increased during the whole sampled period) (13, 56). Additionally, some patients were found to have elevated levels even 1 year after trauma (56).

#### Suggested Serum *t*_1/2_ of NF-L

With the data available, it is not possible to determine a serum *t*_1/2_ for NF-L (13, 56). However, it is evident that this is the protein with the longest *t*_1/2_ of these biomarkers.

## Discussion

This systematic review highlights that serial sampling of different biomarkers in serum results in distinguishably different temporal trajectories in TBI patients. Serum S100B and UCH-L1 levels seem to have the shortest *t*_1/2_ while the serum levels of the biomarkers GFAP and NSE both remain elevated for a prolonged period of time, as compared to S100B and UCH-L1. Even more extended, NF-L appears to have the longest *t*_1/2_ of the biomarkers. However, a specific value could not be identified in the studies, as it continued to increase over the sampling period of 2 weeks. Due to the heterogeneity of included patients, secondary brain injury development, assays used, and sampling frequency, it is impossible to draw any accurate conclusions regarding standardized elimination half-lives after concentration peaks for these proteins, but we believe our effort including effective half-lives provides the best possible attempt to date. Moreover, different sources of biomarkers seem to influence the total serum levels over time, with extracranial contribution being most influential for S100B and NSE, where this has been most extensively studied. Despite these caveats and in contrast to the other biomarkers, S100B and NSE have fully automated clinical assays, making them accessible for routine clinical use. To our knowledge, this is the first systematic review of temporal profiles of biomarkers following TBI, and it could serve as a platform to better assess and compare novel brain biomarkers to be introduced, as well as relate future studies presenting serial sampling of TBI patients.

**Figure 3:**
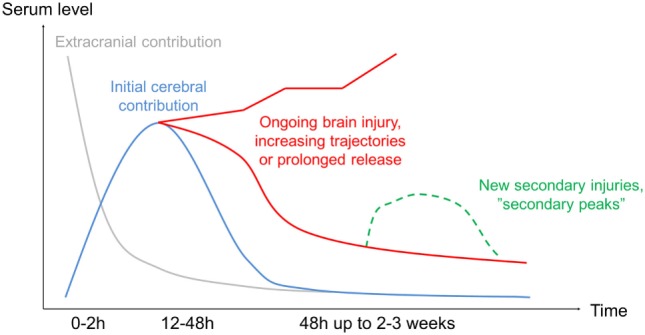
Protein kinetics following injury. Highlighting the estimated temporal profile from trauma if frequently sampled. Initially, there will be a major release of the protein from extracranial sources (gray line), in theory more so if it is present to a larger extent in tissue likely to be injured (i.e., S100B in adipose tissue), even if this contribution generally decreases rapidly. The cerebral contribution (blue line) will continue to increase in serum (for S100B up to 27 h), presumably due to influx from the injured brain. In case of ongoing injury development, the subsequent serum samples may have a prolonged decline or even continue to increase depending on the injury severity (red line). In case of a new injury development, secondary peaks have been shown (green line). While this pattern is most studied for S100B, it applies to some extent to all the biomarkers even if the time frames are different.

Unsurprisingly, the searches that generated the greatest numbers of research articles were that of S100B and NSE. These markers are by far the most studied in TBI but have also been studied in other intracranial conditions, mainly in stroke (118) and to assess brain injuries in patients suffering from circulatory arrest (119). Regarding different aspects of the temporal trajectories, S100B is by far the most studied. It is becoming increasingly clear that temporal changes of S100B in serum are highly dynamic following brain injury (39, 66, 73, 120). Figure 3 is an attempt to better illustrate these changes, and while it is constructed with the dynamics of S100B in mind, it may be generally applicable to the other biomarkers as well, but with different *t*_1/2_. The exception is NF-L, where the *t*_1/2_ is so long that it has not yet even been estimated. The highest levels of S100B are seen early (within 60–120 min after trauma) in patients suffering from multitrauma where bone, adipose tissue, and internal organs [tissues known to express S100B as well (121, 122)] are injured (11, 48). However, these extracranial contributions of S100B will decrease rapidly, as is seen in patients with only multitrauma and without brain injury (48). Jackson et al. estimated these rapid falls of S100B to have a serum “half-life” of 198 min (first sample within 60 min) (39). Townend et al. looked at S100B in mild TBI patients and found that S100B only had an estimated “half-life” of 97 min, even though these samples were acquired a bit later after trauma (CI: 75–136 min, first sample within 240 min following trauma) and all did not have structural injuries (37). In our own experience, the highest level of S100B in serum we have seen was 23.0 µg/L (healthy reference < 0.11 and 1.0–2.0 µg/L usually seen in severe TBI patients) sampled 29 min following trauma from a patient who had fallen from the fifth floor and had severe extracranial injuries as well as intracranial focal mass lesions [patient in study (11)]. The next sample was 6.2 µg/L acquired 6.5 h after trauma, suggesting a “half-life” similar to Jackson et al. of around 3 h. It is import to realize that levels of S100B may represent two processes, where the initial early peak probably represents a more bolus-like dose of S100B assumed predominantly of extracranial origin and is eliminated quickly (Figure 3), here better reflecting its true serum elimination half-life. The second peak, after about 24 h, represents a slow release net sum of influx and outflux of S100B to serum, predominantly from the injured brain and where the slower decay is affected by the continued release, thus the extended *t*_1/2_. This interpretation is supported by our study of moderate-to-severe TBI patients, where we saw that the late 24 (27.2)-h peak is highly related to outcome whereas the initial peak is not (11). We have also modeled the functional kinetics of S100B in moderate-to-severe TBI patients, after excluding this initial “trauma peak” (73). S100B was found to have an expected “brain injury” peak level at around 27 h after injury (38). After that peak, it should drop with an expected rate during the upcoming days (38). If S100B does not follow this trajectory, it could indicate ongoing brain injury (9, 43, 44), resulting in an unfavorable outcome (11). We must stress that the trajectory described here is that of TBI and has not been extensively studied in other contexts. Our experience of several thousand patients in routine clinical use, in for example patients with embolic stroke, is that they can express an extended release, often peaking at day 2–3. The cause of this is not yet understood, but could reflect ongoing penumbral leakage or patterns of recirculation. The presence of secondary peaks of S100B should be highlighted (7, 8, 44–46), as they have been shown to be associated with secondary brain injuries or neurological deterioration in TBI patients. In summary, it is important to understand the kinetic profile of S100B, and its different components when interpreting it as a biomarker of brain injury.

The second most studied protein was NSE. Similar to S100B, a steady decline is generally seen but with a serum *t*_1/2_ longer than for S100B. However, patients with severe injuries may continue to present increasing levels in serum after trauma (47, 48). The general decrease seen for NSE may be delayed in patients with unfavorable outcome or more severe injuries (11, 44), and NSE has been shown to be influenced by extracranial contribution (11, 49), possibly more so than S100B. Another major caveat with NSE is its presence in erythrocytes making serum sampling unreliable if hemolysis is present (50) despite that there are tools attempting to adjust for this (51) and procedures in automatic clinical assays that discard. Similar to S100B, secondary peaks of NSE have been shown in patients with new or progressing injuries (44, 52). In aggregate, NSE behaves similar to that of S100B in serum, albeit it appears to have a longer contextual half-life in serum of about 48 h and has larger influence from extracranial sources.

Serial sampling of GFAP has been less commonly studied in TBI, but interest is increasing, presumably due to GFAP’s superior brain specificity (41). The serum *t*_1/2_ levels of GFAP are extended, as compared to S100B, presumably at around 24–48 h in severe TBI patients and thus similar to NSE. This prolonged increase in serum levels may prove to be beneficial for diagnostic screening of intracranial lesions in milder TBI, being more detectable >6 h after injury, as compared to S100B (53, 54). However, it appears to lack granularity to detect more rapidly changing trajectories as seen when serially sampling proteins with shorter effective half-lives, such as S100B and UCH-L1. This may explain why only a limited amount of studies report secondary peaks of GFAP (12). A long *t*_1/2_ will make it difficult to use in assessment of treatment efficacy and monitoring, as it would in theory provide a delayed treatment response and show a blunted concentration. Despite this, a delayed decrease or continued release of GFAP is seen in patients with unfavorable outcome (53, 55–57). Patients with mass lesions appear to have higher levels of GFAP in serum as compared to more diffuse injuries (12, 58), especially in combination with lower levels of UCH-L1 (used in a glial:neuronal ratio) (59). While GFAP has been seen to increase in patients with extracranial trauma and without brain injury (60), reports of serial sampling in multitrauma populations are scarce (108), but as the protein is so much more brain specific as compared to S100B (Table S1 in Supplementary Material; Table 6) (41), available data suggest that extracranial contribution over time to be relatively low. In summary, GFAP seems to have longer *t*_1/2_ half-life than S100B, of about 24–48 h, which might prove beneficial for screening purposes if a patient is sampled late after ictus, but might decrease accuracy to detect and separate novel lesions and monitor ongoing events.

**Table 6:** Characteristics of the selected protein biomarkers.

Protein	Molecular weight	Primary origin	Automated assay	Extracranial contribution	Effective serum half-life	Clinical relevance
S100B	9–11 kDa	Astrocytes	Available	Relatively high	Short (hours up to 24 h)	+ Effective for serial sampling and monitoring purposes, can detect secondary deterioration. + Well validated in the literature. − Extracranial contribution lowers its potential early after multitrauma.
Neuron-specific enolase	47 kDa	Neurons	Available	Relatively high	Long (24 h–3 days)	+ Rather well validated in the literature, have been shown to detect secondary deterioration. − Hemolysis leads to high levels in serum. − Extracranial contribution lowers its potential in multitrauma. − Relatively long effective half-life limits the potential for monitoring.
Glial fibrillary acidic protein	50 kDa	Astrocytes	Not available	Very low	Long (24 h–2 days)	+ Low extracranial contribution. + Rather well validated in the literature, have been shown to detect secondary deterioration. − Relatively long effective half-life limits the potential for monitoring.
Ubiquitin carboxy-terminal hydrolase L1	25 kDa	Neurons	Not available	Low	Short (hours up to 12–24 h)	+ Low extracranial contribution. + Should be effective for serial sampling and monitoring purposes because of short effective half-life. − Limited validation in the literature, but has been shown to detect secondary deterioration.
Neurofilament light	68–70 kDa	Neurons	Not available	Very low	Very long (3 weeks?)	+ Low extracranial contribution. − Very long effective half-life limits the potential for monitoring. − Limited validation in the literature.

*Illustration of some of the protein characteristics. Primary origin indicates which cell in the central nervous system contain highest amount of the specific protein. Molecular weight is the size of the protein in kilo Dalton and the primary cellular origin is the cells with highest amount of expressed concentration in the central nervous system. If an automated clinical assay platform is available, it is indicated. Extracranial contribution is an aggregate from Table S1 in Supplementary Material, indicating how much protein and mRNA that is expressed outside the central nervous system. Serum effective half-life is an aggregate of the findings in this study. Clinical relevance is exemplified*.

Brophy et al. analyzed serum levels of UCH-L1 with a high sampling frequency and established its serum functional half-life to be in the vicinity of 7–9 h (115). They also noted it to differ between severe and mild injury. Moreover, similar to the other markers, they discovered some individual patients with secondary increases (29, 61). This decrease was slower in patients with more severe injuries and worse outcomes (29, 53), also analogous with the other biomarkers. Interestingly, and in contrast to GFAP, diffuse injury seems to lead to higher levels of UCH-L1 as compared to focal mass lesions (12). UCH-L1 is more brain specific than S100B (41), but data indicate that it is also significantly increased in patients with extracranial injuries (60). In aggregate, UCH-L1 appears to have a relatively short functional half-life, similar to that of S100B, but needs further studies to elucidate its temporal profile following trauma as well as more robust associations with extracranial injuries.

The protein NF-L is the least studied in a temporal context, presumably because no commercial assay is available at present. The two studies investigating this biomarker in TBI populations noted that serum levels of NF-L continually increased the first week(s) after injury (13, 34). There are no *in vivo* studies that have appropriately assessed the serum half-life of NF-L, but an *in vitro* report suggests that it may be as long as 3 weeks (62), which could be possible looking at available data. Surprisingly, it was found elevated even at up to a year in some patients (34), perhaps indicating ongoing pathology. Neurofilament heavy (NF-H) is another, similar, axonal protein that has been studied in TBI and shows similar trends with continually increasing serum levels the first days after trauma (63). A case series suggests that NF-L may aid in assessment of diffuse axonal injury (64), and a study indicates that it adds independent information in outcome prediction models, in addition to S100B (13). As reliable assays become more readily available, there might be a growing interest in this marker, which could reflect an ongoing neuroinflammatory pathology, distinctly different to the others studied here. However, considering its serum dynamics, it would probably provide little information on the rapid development of novel intracranial lesions the first week in the NICU but would instead be of greater interest in later, more chronic phases of TBI.

**Figure 4:**
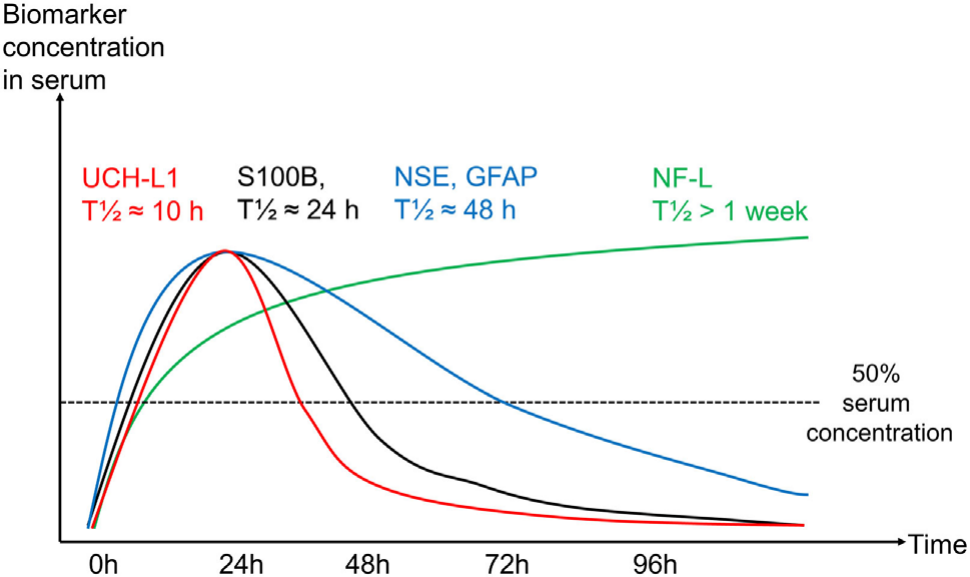
Protein kinetics for each protein in serum over time. Graph illustrating how the influence of an increasing effective half-life results in a serum sample in an uneventful traumatic brain injury (TBI) (without secondary deterioration) for ubiquitin carboxy-terminal hydrolase L1 (UCH-L1) (red), S100B (black), neuron-specific enolase (NSE)/glial fibrillary acidic protein (GFAP) (blue), and neurofilament light (NF-L) (green). Biomarker concentration in serum on *y*-axis and time in hours on *x*-axis. Note that these are estimates based on the knowledge of S100B kinetics in serum, current literature makes it difficult to illustrate more accurate trajectories over time for the other proteins.

In Table 6, we present an aggregate of our findings on these markers. Our review finds the effective serum half-lives of these biomarkers to be similar in ranges to those suggested by a recently published narrative review of the field where, among others, S100B, NSE, GFAP, and UCH-L1 were included (65). In contrast to that narrative review, we have focused on the serum compartment, attempting to systematically interpret information on effective half-lives from all available studies. While it was impossible to conduct a proper meta-analysis, we summarized the available literature in histograms for S100B (Figure 2A), NSE (Figure 2B), GFAP (Figure 2C), and UCH-L1 (Figure 2D), where it is possible to see that S100B and UCH-L1 have the most amount of studies that indicate a shorter effective serum half-life while GFAP and NSE exhibit relatively longer serum *t*_1/2_ (Figure 4).

Many factors could affect the contextual half-lives of the studied biomarkers, and we acknowledge that the serum effective half-lives provided here might still be inaccurate. While we excluded studies without structural imaging, we did not look at lesion progression as only one study properly reported this (9), something we believe will influence trajectories of serially sampled proteins. The association between lesion size and biomarker levels was also barely mentioned (66). Together, these conditions make it difficult to accurately generate a precise half-life due to the constant influx/efflux of the proteins to the serum compartment (20). Furthermore, some studies suggest peak times of biomarkers as related to time after trauma (73, 102, 112, 115). Currently, S100B is the only protein where attempts have been made to model the temporal contextual kinetics following injury (73), something that also needs to be performed for the others. We believe that all of these serum proteins, except for NF-L, peak relatively early in serum and any unexpected prolonged release might not be a natural progression but could indicate deterioration or ongoing damage in some way. Moreover, it is unknown to what extent these proteins are expressed after trauma, also potentially affecting levels. Additionally, extracranial injuries give rise to some of these proteins in serum as well, primarily NSE and S100B, resulting in altered serum dynamics (11, 48). While UCH-L1 and GFAP have been shown to be increased in serum of non-head injured patients as well (123), they are, together with NF-L, more brain specific (122). We show this in Table S1 in Supplementary Material, where we have gathered protein and mRNA expression (in tissues usually injured in multitrauma patients). In aggregate, extracranial contribution and injury progression need to be better assessed in future studies as it will affect contextual kinetics.

Several articles mentioned refer to the term “serum half-life” when trying to describe the temporal profiles following TBI. We believe that this description is inaccurate as we are not looking at protein decay in a single space; instead it is probably a combination of influx and efflux between bodily compartments with ongoing release from the injured brain, where actual clearance is one of many actors (20). Thus, we have used the term “effective serum half-life” to describe that concentration dynamics is presumably more accurate as this is not a process with constant decay (i.e., as is true in theory for a biological half-life).

We could not find any signs that younger patients should have a different temporal profile than adults for these proteins. Instead, and as can be seen in the tables, biomarker dynamics in serum appear to be correlated with injury severity. However and notably, pediatric populations were not nearly as frequently studied as adult TBI patients. The reference levels for healthy pediatric populations (especially the first year) of S100B and NSE have both been shown to be significantly higher than for adults (factor ×2–4) (124, 125). Nevertheless, during traumatic conditions, similar trajectories to adults are seen in pediatric populations, and serum dynamics are likely a marker of injury severity and progression not requiring separate reference levels per age group. In aggregate, age does not seem to play a major role in biomarker serum dynamics, but is not as well studied in pediatric populations as compared to adults.

The literature varies greatly in terms of the sampling frequency chosen. This may be problematic when attempting to determine the detailed kinetics. For many studies, the generally long sampling intervals chosen severely limit our knowledge of its early behavior. The choice of optimal sampling frequency to ensure faithful replication of a time series has received extensive investigation in information theory (126). In essence, the sampling frequency must be chosen to be at least twice the characteristic frequency of the signal. In other words, if changes are expected over a particular time period, then the sampling interval must be at most half of this and preferably more frequent still. Thus, we suggest that future prospective studies consider the following issues:

– Sampling frequency: It is best advised to perform a high initial sampling frequency to accurately map trajectories over time (proteins like S100B/UCH-L1 needs a higher frequency than NF-L). If early detection is the goal, then a tapered strategy may help identify peak with early frequent sampling.
– Relations to imaging: A high frequency of imaging will best aid association of biomarker trajectories with potential injury progression. Current imaging modalities in practice limit the frequency possible.
– Relations to other monitoring: High frequency multimodal monitoring (metabolism, oxygenation, intracranial pressure, etc.) may help associate biomarker trajectories with potential secondary insults/deterioration.
– TBI population: We suggest to identifying and studying TBI cohorts that are clinically and pragmatically definable such as NICU TBI patients, thus aiming to understand a biomarker in the context of the population it is expected to be clinically used in.
– Blinding: Serum biomarkers should be analyzed in retrospect or blinded as to not influence treatment strategies in a study setting.
– Analysis method: If possible, use a well validated assay, preferably with industrial-level calibration.

Readily available and reliable assays are crucial if protein biomarkers are to find routine clinical application. To date, automated assays with industrial calibrations are only available for S100B and NSE, and this makes it possible to provide reliable analyses in less than 20 min from sampling. Until this is widespread, it will be difficult for the other proteins to reach everyday use as these assays (i.e., ELISAs) take around 6 h or more to run. Moreover, without proper automatic assays with regular, standardized calibrations, there is a risk for greater inter- and intravariability between samples and studies, as has been seen between ELISA methods for S100B (67). Actually, we noticed that several studies that did use ELISA for NSE and S100B showed a different release pattern after trauma with consisting higher levels over time, as compared to the automated assays, resulting in longer serum half-lives (66, 68, 69). This is worrying as it may imply that the assays (and thus the studies) are not as comparable as has been suggested, presumably due to different antibodies used or different lower levels of detection, stressing the need for standardized testing in the field. Likely, while we did not particularly focus on the exact levels but on the temporal profiles, they would also be affected by this.

### Limitations

We aimed to perform a meta-analysis of the data collected but realized that this was not possible primarily due to the use of different assays, differences in sampling time and heterogeneous patient populations. Instead, we have listed estimates of serum half-lives by assessing graphs, tables, and data sets from previous studies, which we believe generates the best possible current estimates of the effective serum half-lives of these proteins after TBI.

Studies of S100B and NSE are more frequent than studies analyzing UCH-L1 and NF-L. Results concerning the proteins with little data should be interpreted with more caution. In the case of NF-L, only two studies were available and the uncertainty is large here. Indicative of this is that in contrast to the findings on NF-L, NF-H has been found to have a short half-life in mild TBI *t*_1/2_ (48–72 h) (127). As these two components may have similar half-lives and as no mild TBI study is available for NF-L, it is possible that NF-L has a shorter *t*_1/2_ in this population as well.

It is possible that several papers coming from the same research centers contain, to some extent, the same patients several times. We have not been able to adjust for this possibility. We mention all studies in the tables but focus on the largest patient cohort from each group in the Section “Discussion.”

While we have acknowledged a difference in sampling frequency between basically all studies, one further issue is that a majority of studies report sampling since admission, not from the actual trauma time. As the dynamics for a protein such as S100B differs substantially in time the first 24 h (38), having the exact trauma time reported is essential to generate adequate models of biomarker release.

## Conclusion

It is increasingly apparent that the dynamic behavior of serum TBI biomarkers varies greatly and an appreciation of this is critical for their interpretation as markers of tissue fate. The initial intracranial injury, potential extracranial trauma as well as injury progression and the occurrence of secondary injuries will influence the biomarker temporal profile. Unfortunately, while serial sampling is common in studies, few adequately comment on the temporal profiles of the analyzed proteins and even less address what sampling frequency is needed to capture information content. From a clinical perspective, and with the aim of using biomarkers as ongoing monitors of TBI patients, proteins with shorter serum availability such as S100B and UCH-L1 may be advisable as compared to proteins such as NSE, GFAP, and NF-L, as the longer peak times and half-times may lag detection of secondary harmful events. Moreover, brain specificity of the proteins should be taken into account and the need for fast, reliable assays is the definite current rate-limiting step in research that may lead biomarkers to clinical use. Further prospective research on the contextual kinetics of protein biomarkers is urgently warranted if their full diagnostic potential is to be realized.

## Author Contributions

ET, DN, AE, FZ, AB, B-MB, AH, SM, and DM designed and planned the study; drafted the manuscript which all authors read and approved. ET conducted the systematic review with the help from FZ.

## Conflict of Interest Statement

The authors declare that the research was conducted in the absence of any commercial or financial relationships that could be construed as a potential conflict of interest.
