# Supplementary material for: Serial Sampling of Serum Protein Biomarkers for Monitoring Human Traumatic Brain Injury Dynamics: A Systematic Review
Source: Front Neurol. 2017 Jul 3;8:300. doi: 10.3389/fneur.2017.00300 (PMC5494601; doi:10.3389/fneur.2017.00300)
Supplement: Supplementary file 3 [file Data_Sheet_1.DOC]

**Biomarker Systematic Review - MEDLINE Search Strategy**

1. traumatic brain injury.mp. [mp=ti, ab, hw, tn, ot, dm, mf, dv, kw, fs, bt, id, cc, nm, kf, px, rx, an, eu, pm, ui]

2. traumatic brain injury.tw.

3. TBI.mp.

4. TBI.tw.

5. brain injury.mp.

6. brain injury.tw.

7. head injury.mp.

8. head injury.tw.

9. head trauma.mp.

10. head trauma.tw.

11. cerebral trauma.mp.

12. cerebral trauma.tw.

13. brain trauma.mp.

14. brain trauma.tw.

15. cerebral injury.mp.

16. cerebral injury.tw.

17. craniocerebral trauma.mp.

18. craniocerebral trauma.tw.

19. cranial trauma.mp.

20. cranial trauma.tw.

21. cranial injury.mp.

22. cranial injury.tw.

23. 1 or 2 or 3 or 4 or 5 or 6 or 7 or 8 or 9 or 10 or 11 or 12 or 13 or 14 or 15 or 16 or 17 or 18 or 19 or 20 or 21 or 22

24. blood.mp

25. serum.mp

26. plasma.mp

27. 24 or 25 or 26

Biomarker input for the different searches:

S100B:

S100B.mp or
S100 Calcium Binding Protein beta Subunit.mp or
S100b.mp or
S100 beta.mp or
S100beta.mp or
S100.mp or
S-100.mp or

NSE:

Nervous System-Specific Enolase.mp or
Enolase, Nervous System-Specific.mp or
Nervous System Specific Enolase.mp or
System-Specific Enolase, Nervous.mp or
Neuron-Specific Enolase.mp or
Enolase, Neuron-Specific.mp or
Neuron Specific Enolase.mp or
Enolase 2.mp or
NSE.mp or

GFAP:

GFAP.mp or
Glial Fibrillary Acidic Protein.mp or
Glial Fibrillary Astrocytic Protein.mp or

Mesh UCH-L1:

Ubiquitin carboxyl-Terminal Hydrolase L-1.mp or
UCH-L1.mp or
UCHL1.mp or

Mesh NF-L:

Neurofilament Light.mp or
neurofilament protein light.mp or
light neurofilament protein.mp or
Nefl protein.mp or
NFL protein.mp or
NF68 protein.mp or
CMT2E protein.mp or
neurofilament.mp or
NF68 protein.mp or
Neurofilament triplet L protein.mp or
neurofilament, light polypeptide 68kDa protein.mp or
CMT1F protein.mp or
NF-L.mp or
NFL.mp

Search string was “23 and 27” + the different biomarker strings.
