# Supplementary material for: Serial Sampling of Serum Protein Biomarkers for Monitoring Human Traumatic Brain Injury Dynamics: A Systematic Review
Source: Front Neurol. 2017 Jul 3;8:300. doi: 10.3389/fneur.2017.00300 (PMC5494601; doi:10.3389/fneur.2017.00300)
Supplement: Supplementary file 4 [file Data_Sheet_2.DOCX]

Supplementary Material

Serial sampling of serum protein biomarkers for monitoring human traumatic brain injury dynamics: A systematic review

*** Eric Peter Thelin, Frederick Adam Zeiler, Ari Ercole, Stefania Mondello, András Buki, Bo-Michael Bellander, Adel Helmy, David K Menon, David W Nelson**

**Correspondence:** Eric P Thelin: eric.thelin@ki.se

# Supplementary Data

Supplementary Data 1 – PRISMA checklist

**Supplementary Data 1.** Reported data from this review using the Preferred Reporting Items for Systematic Reviews and Meta-Analyses (PRISMA) checklist.

Supplementary Data 2 – Search string strategy used on MEDLINE

**Supplementary Data 2.** The search strings using MEDLINE and EMBASE. Similar search strategy utilized for SCOPUS. All possible MESH-terms were used for the different biomarkers.

# Supplementary Figures and Tables

For more information on Supplementary Material and for details on the different file types accepted, please see [here](http://home.frontiersin.org/about/author-guidelines#SupplementaryMaterial).

## Supplementary Figures

Supplementary Figure 1 – NSE inclusion flowchart


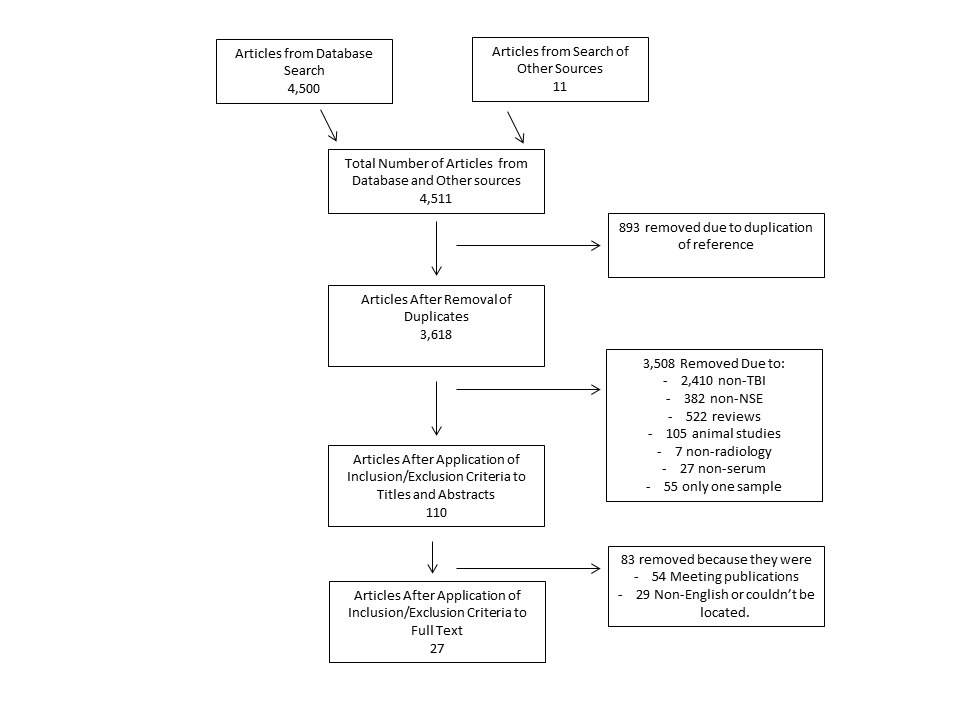


**Supplementary Figure 1.** Illustrating the selection of articles with serial sampling of NSE.

Supplementary Figure 2 – GFAP inclusion flowchart


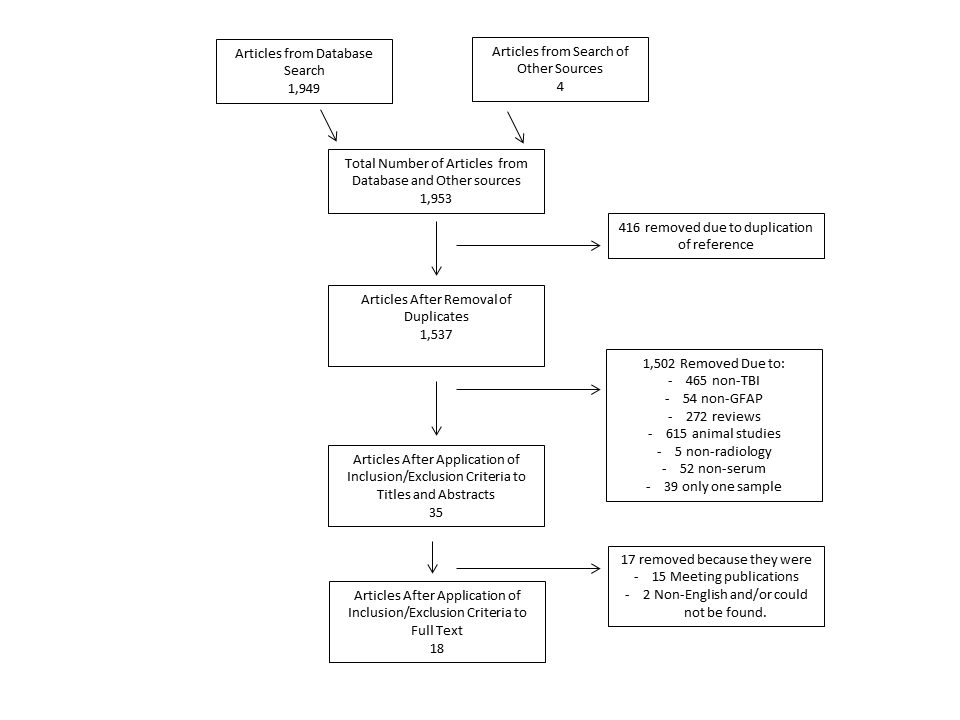


**Supplementary Figure 2.** Illustrating the selection of articles with serial sampling of GFAP.

Supplementary Figure 3 – UCH-L1 inclusion flowchart


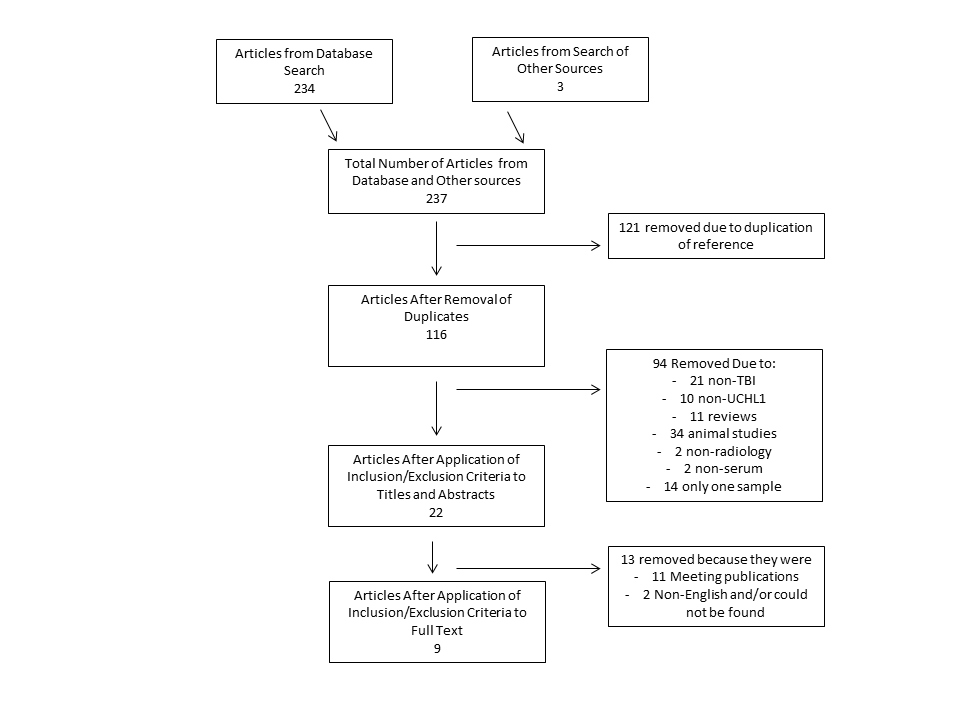


**Supplementary Figure 3.** Illustrating the selection of articles with serial sampling of UCH-L1.

Supplementary Figure 4 – NF-L inclusion flowchart


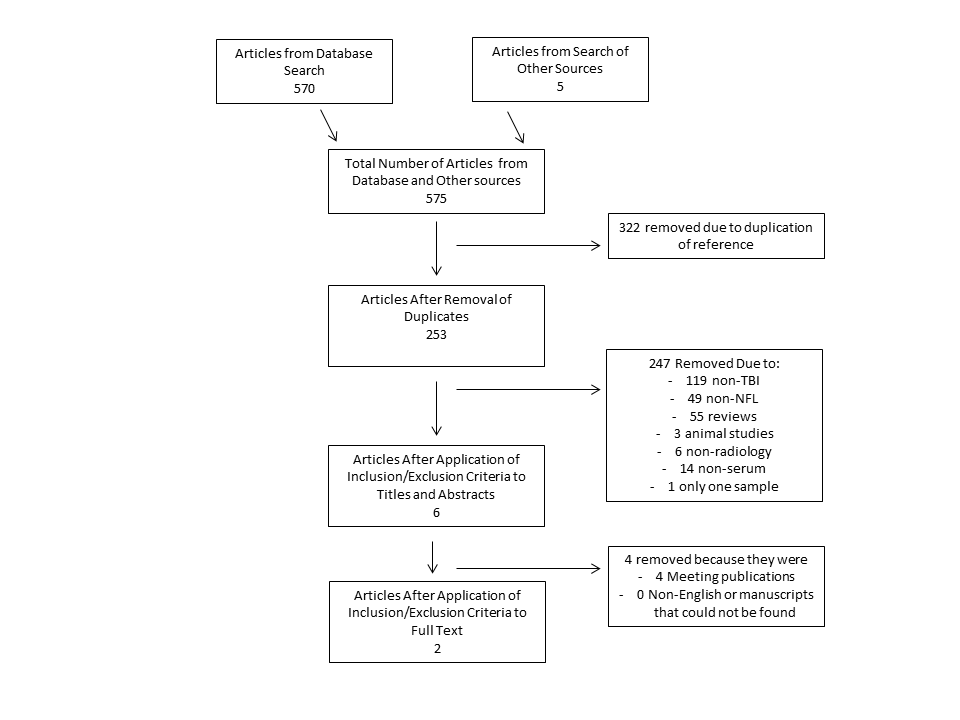


**Supplementary Figure 4.** Illustrating the selection of articles with serial sampling of NF-L.

| **Biomarker** | **Tissue also expressing protein (according to Human Protein Atlas)** | **mRNA more frequent in brain vs adipose tissue** | **mRNA more frequent in brain vs skeletal muscle** | **mRNA more frequent in brain vs bone** | **mRNA more frequent in brain vs skin** |
| --- | --- | --- | --- | --- | --- |
| S100B | Colon (ganglion), breast (myoepithelial cells), adipose tissue, peripheral nerves, skin | 1.4x | 2,093x | 171x | 41x |
| NSE | Pancreas (islets of Langerhans), erytrocytes, colon (ganglion), peripheral nerves | 45x | 360x | 77x | 30x |
| GFAP | Not detected | 375x | 5,139x | 19,600x | * |
| UCH-L1 | Pancreas (islets of Langerhans), colon (ganglion), kidney, testis, peripheral nerves | 23x | 161x | 650x | 182x |
| NF-L | Not detected | 1,415x | * | * | 2,829x |

Supplementary Table 1 – Extracranial distribution and expression of the protein biomarkers

**Supplementary Table 1.** Brain specificity uses the “tissue specific score” from Sjöstedt et. al [1] and/or the Human Protein Atlas® (proteinatlas.org), brain tissue mRNA expression divided by mRNA expression for adipose, skeletal muscle, bone and skin tissue commonly injured in traumatic conditions. An asterisk (*) indicates that the specific protein mRNA is not expressed at all in the analyzed tissue.

[1] E. Sjostedt, L. Fagerberg, B.M. Hallstrom, A. Haggmark, N. Mitsios, P. Nilsson, F. Ponten, T. Hokfelt, M. Uhlen, and J. Mulder, Defining the Human Brain Proteome Using Transcriptomics and Antibody-Based Profiling with a Focus on the Cerebral Cortex. PLoS One 10 (2015) e0130028.
